# Supplementary material for: A multi-ethnic polygenic risk score is associated with hypertension prevalence and progression throughout adulthood
Source: Nat Commun. 2022 Jun 21;13:3549. doi: 10.1038/s41467-022-31080-2 (PMC9213527; doi:10.1038/s41467-022-31080-2)
Supplement: Supplementary file 1 — Supplementary Information [file 41467_2022_31080_MOESM1_ESM.pdf]

A multi-ethnic polygenic risk score is associated with hypertension prevalence and development throughout adulthood:

Supplementary Information

Kurniansyah et al.

Supplementary Note 1: Computing PRS using previously-reported methods that model pleiotropy 3

Supplementary Note 2: Global ancestry inference in TOPMed....5

Supplementary Tables....6

Supplementary Table 1: External GWAS used for hypertension PRS construction in secondary analysis....6

Supplementary Table 2: Characteristics of the TOPMed stage 1 (BioMe) training dataset....6

Supplementary Table 3: Characteristics of TOPMed stage 2 dataset participants. 7

Supplementary Table 4. Tuning parameters and SNP counts in primary and secondary PRS trained using stage 1 dataset....8

Supplementary Table 5. Results from association analysis of the number of individuals in longitudinal BP categories and HTN-PRS deciles....9

Supplementary Table 6. Characteristics of the stage 3 CARDIA dataset....10

Supplementary Table 7. Characteristics of the stage 4 MGB Biobank dataset....11

Supplementary figures....12

Supplementary Figure 1: Association of primary PRS with hypertension in the stage 1 dataset across compared tuning parameter selection criteria....12

Supplementary Figure 2. Association of primary and secondary PRS with hypertension in the BioMe stage 1 dataset across tuning parameter selection criteria....13

Supplementary Figure 3. Distribution of longitudinal categories of BP stratified by race/ethnic background....14

Supplementary Figure 4. Distribution of longitudinal categories of BP stratified by age....15

Supplementary Figure 5: Correlation heatmap between Selected CV PRS by phenotype and PRSsum stratified by race/ethnic background....16

Supplementary Figure 6: Association of HTN-PRS with hypertension measures across race/ethnicities, with group-specific PRS scaling. 17

Supplementary Figure 7. Risk of hypertension in individuals in high deciles of the HTN-PRS compared to those in low deciles of the HTN-PRS. 18

Supplementary Figure 8. Age-stratified associations of HTN-PRS with prevalent and incident hypertension....19

Supplementary Figure 9: Benchmarking the risk of HTN-PRS against other risk factors....20

Supplementary Figure 10: Race/ethnicity stratified associations of the HTN-PRS with outcomes in the MGB Biobank......21
Supplementary Figure 11: LD-Score regression based estimates of heritability and genetic correlations between BP phentoypes......22
......22
Supplementary Figure 12: Comparisons of the associations of the HTN-PRS and pleiotropy-based PRS with hypertension in the stage 1 dataset......23
Supplementary Figure 13: Comparisons of the associations of the HTN-PRS and pleiotropy-based PRS with hypertension in the stage 2 dataset......24
Supplementary Figure 14: BP trait PRS distribution stratified by groups defined by genetic ancestry......25
Supplementary Figure 15: Association of HTN-PRS with hypertension measures across groups defined by high proportions of genetic ancestries......26
Supplementary Note 3: Study descriptions......27
BioMe......27
ARIC......28
CHS......30
CARDIA......32
FHS......33
GENOA......35
HCHS/SOL......36
JHS......38
WHI......40
MESA......41
MGB Biobank......44
Supplementary Note 4: TOPMed and CCDG acknowledgements......48
Supplementary Note 5: TOPMed consortium authors......50
References......53

Supplementary Note 1: Computing PRS using previously-reported methods that model pleiotropy

We applied two approaches to model pleiotropy [1,2] to compute PRS for hypertension using summary statistics from GWAS of SBP, DBP, and hypertension. With both approaches, we created two potential PRS: one using newly-developed summary statistics that account for pleiotropy in each SNP separately, and second by weighting and summing PRS constructed for the three phenotypes, with weighting performed by accounting for pleiotropy. All implementations required the estimation of heritability of the three phenotypes and the genetic correlation between them, methods for estimation of heritabilities and genetic correlations are described below.

Estimation of heritabilities and genetic correlations using LD-Score regression:

We followed the recommendations of these of the publications reporting the pleiotropy-accounting methods and used LD-Score regression [3] with the `ldsc` (v1.0.1; https://github.com/bulik/ldsc) package to compute heritabilities and genetic correlations. As the three GWAS used were from a multi-ancestry analysis, we used our TOPMed dataset as the reference panel when computing these estimates. We limited the number of SNPs used by `ldsc` to ~1 million SNPs by removing all SNPs with any missing value in the sample, and clumping using parameters R^2=0.6 and distance=500Kb. Estimated heritabilities and genetic correlations are provided in Supplementary Figure 10.

MTAG-GWAS and SMTpred-GWAS PRS:

To create summary statistics for hypertension while leveraging pleiotropy with SBP and DBP, we used MTAG (Multi trait analysis GWAS)[1] and SMTpred [2]. The two approaches rely on different models, but the end goal is the same. Notably, both approaches account for LD between SNPs to calculate the new summary statistics using LD scores. Next, we constructed PRS, denoted by MTAG-GWAS and SMTpred-GWAS based on the new summary statistics using PLINK v2.0 [4].

MTAG-SBLUP and SMTpred-wSBLUP PRS:

Based on MTAG results above, we created another PRS using GCTA v1.91.1 [5] as an SBLUP. This score is called MTAG-SBLUP. We also generated SBLUPs using GCTA for each of the hypertension, SBP, and DBP traits (based on the original summary statistics), and combined them using weights informed by the pleiotropy-based models of SMTpred. This PRS is called SMTpred-wSBLUP. These approaches follow the recommendations of the respective manuscripts reporting MTAG and SMTpred, with the different that we used SBLUP rather than LDPred PRS for MTAG-SBLUP.

Results:

Supplementary Figures 12 and 13 compares the association of the HTN-PRS (PRSsum based on Selected CV-PRS) to the associations of the four PRS constructed using pleiotropy-aware methods with hypertension the stage 1 and stage 2 datasets. The HTN-PRS had better performance. Further discussion is provided in the main manuscript.

Supplementary Note 2: Global ancestry inference in TOPMed

Ancestry inference was performed by the TOPMed Informatics Research Center (IRC). First, local ancestry was inferred using RFMix [6], with default parameter settings except the following option: --node-size=5. Then, global ancestry was computed as for each participant as a weighted average of the ancestries in inferred local ancestry intervals. The reference panel used was the Human Genome Diversity Panel (HGDP) downloaded from the Stanford HGDP website http://hagsc.org/hgdp/files.html. Genomic coordinates were lifted over from genome build 37 to build 38. The 53 HGDP populations were merged into 7 super-populations: Sub-Saharan Africa, Central and South Asia, East Asia, Europe, Native America, Oceania, Middle East. Local ancestry inference was performed in two versions. First, for samples available in TOPMed freeze 6, RFMix V1 was used, and local ancestry was inferred for the autosomes only. Later, for samples participating only in freeze 8 (but not in freeze 6), and for the X-chromosome, local ancestry inference was performed using RFMix V2.

Supplementary Tables

Supplementary Table 1: External GWAS used for hypertension PRS construction in secondary analysis

GWAS name	Reference	Trait	Sample Size	Population
FinnGen	No manuscript, summary statistics were downloaded from the FinnGen biobank's repository of association results. See https://www.finngen.fi/en/about	HTN	135,638 (34,257 cases, 101,358 controls)	Finnish-European
BBJ	PMID:29403010 [7]	SBP; DBP	SBP (136,597); DBP (136,615)	Japanese
UKBB+ICBP	PMID:30224653 [8]	SBP; DBP	SBP (757,601); DBP (757,601)	European
Pan-UKBB	No manuscript, downloaded from https://pan.ukbb.broadinstitute.org	SBP; DBP	SBP (453,005); DBP (453,010)	African (1.40 %), Admix American (0.21 %), European (86.94 %), Central/South Asian (1.81 %), Middle Eastern (0.33%), East Asian (0.54%)

The table provides GWAS source, study population as reported by the manuscript or repository reporting the GWAS, and number of participants used to generate summary statistics.

Supplementary Table 2: Characteristics of the TOPMed stage 1 (BioMe) training dataset

Characteristics	African-American	Asian-American	European-American	Hispanic-American	Others
N	2803	151	2506	4187	667
Sex (%) F	1864 (66.5)	84 (55.6)	1198 (47.8)	2616 (62.5)	281 (42.1)
Age (mean (SD))	55.99 (12.50)	53.14 (13.52)	60.42 (13.96)	57.72 (13.18)	57.51 (13.10)
Current smoke (%) Yes	658 (23.6)	10 (6.6)	240 (9.6)	741 (17.8)	94 (14.2)
Antihypertensive medication (%) Yes	1589 (56.7)	48 (31.8)	807 (32.2)	2200 (52.5)	255 (38.2)
BMI (mean (SD))	30.46 (8.20)	24.30 (3.85)	26.58 (7.36)	29.24 (6.50)	27.61 (10.35)

SBP (mean (SD))	145.03 (28.57)	129.93 (27.36)	130.34 (23.75)	139.36 (28.04)	133.38 (24.78)
DBP (mean (SD))	82.42 (15.76)	73.71 (14.97)	73.68 (13.14)	78.04 (14.53)	75.79 (13.39)
Hypertension (%) Yes	2222 (79.3)	85 (56.3)	1477 (58.9)	3084 (73.7)	420 (63.0)

Supplementary Table 3: Characteristics of TOPMed stage 2 dataset participants.

Characteristics	African-American	Asian-American	European-American	Hispanic-American
N	8822	794	22701	6718
Sex (%) F	6004 (68.1)	493 (62.1)	16395 (72.2)	4168 (62.0)
Cohort (%)				
• ARIC	1324 (15.0)	0 (0.0)	6000 (26.4)	0 (0.0)
• CHS	692 (7.8)	0 (0.0)	2765 (12.2)	24 (0.4)
• FHS	0 (0.0)	0 (0.0)	3102 (13.7)	11 (0.2)
• GENOA	1069 (12.1)	0 (0.0)	0 (0.0)	0 (0.0)
• HCHS_SOL	0 (0.0)	0 (0.0)	0 (0.0)	5357 (79.7)
• JHS	3211 (36.4)	0 (0.0)	0 (0.0)	0 (0.0)
• MESA	1096 (12.4)	599 (75.4)	1856 (8.2)	1019 (15.2)
• WHI	1430 (16.2)	195 (24.6)	8978 (39.5)	307 (4.6)
Year between visit (mean (SD))	4.66 (1.03)	4.45 (0.83)	4.81 (1.62)	5.71 (1.00)
Age at baseline (mean (SD))	58.72 (11.29)	62.44 (9.78)	59.97 (12.73)	50.76 (13.46)
Current smoke at baseline (%) Yes	1630 (18.5)	37 (4.7)	4463 (19.7)	1310 (19.5)
Antihypertensive medication at baseline (%) Yes	4637 (52.8)	239 (30.1)	6908 (30.5)	1532 (23.0)
BMI at baseline (mean (SD))	30.92 (6.69)	24.29 (3.62)	27.34 (5.34)	30.06 (6.10)
SBP at baseline (mean (SD))	139.20 (22.58)	131.18 (24.70)	130.80 (22.25)	127.64 (21.29)
DBP at baseline (mean (SD))	82.24 (11.61)	76.90 (12.09)	76.75 (11.35)	76.51 (11.84)
Hypertension at baseline (%) Yes	6711 (76.5)	452 (57.5)	13546 (60.1)	3422 (51.7)
Antihypertensive medication at follow-up(%) Yes	4767 (66.1)	305 (41.8)	8146 (39.4)	2575 (39.5)
BMI at follow-up (mean (SD))	31.19 (6.74)	24.29 (3.68)	27.82 (5.55)	30.31 (6.20)
SBP at follow-up (mean (SD))	141.33 (22.28)	131.41 (23.29)	132.07 (21.73)	130.80 (22.64)
DBP at follow up (mean (SD))	81.71 (11.50)	75.85 (11.25)	76.25 (11.08)	77.12 (11.97)
Hypertension at follow-up (%) Yes	5972 (83.3)	438 (60.8)	13034 (63.8)	3860 (59.6)

Supplementary Table 4. Tuning parameters and SNP counts in primary and secondary PRS trained using stage 1 dataset

Study	Threshold	R2	Distance	# SNP
**Genome-wide Significant**				
Diastolic BP				
BBJ	5.00E-08	0.1	1000kb	46
MVP				86
Pan UKBB				1319
UKBB+ICBP				1730
BBJ+MVP+Pan UKBB				101
Systolic BP				
BBJ	5.00E-08	0.1	1000kb	67
MVP				144
Pan UKBB				1235
UKBB+ICBP				1602
BBJ+MVP+Pan UKBB				166
Hypertension				
FINNGEN	5.00E-08	0.1	1000kb	15
Pan UKBB				582
FINNGEN+Pan UKBB				382
**Selected CV-PRS**				
Diastolic BP				
BBJ	5.00E-01	0.3	250kb	245160
MVP	1.00E-01	0.3	250kb	149559
Pan UKBB	4.00E-01	0.1	1000kb	291655
UKBB+ICBP	2.00E-01	0.1	1000kb	131193
BBJ+MVP+Pan UKBB	2.00E-01	0.1	250kb	253855
Systolic BP				
BBJ	1.00E-02	0.2	1000kb	10280
MVP	1.00E-02	0.2	1000kb	24807
Pan UKBB	1.00E-05	0.1	1000kb	3291
UKBB+ICBP	1.00E-01	0.1	1000kb	87939
BBJ+MVP+Pan UKBB	5.00E-01	0.3	2500kb	444645
Hypertension				

FINNGEN	1.00E-02	0.1	500kb	14215
Pan UKBB	3.00E-01	0.1	250kb	234228
FINNGEN+Pan UKBB	1.00E-04	0.1	1000kb	2486
**Selected PVAL-PRS**				
Diastolic BP				
BBJ	5.00E-01	0.3	500kb	243785
MVP	1.00E-01	0.3	1000kb	147123
Pan UKBB	1.00E-01	0.2	250kb	186307
UKBB+ICBP	1.00E-02	0.1	1000kb	28354
BBJ+MVP+Pan UKBB	1.00E-01	0.1	1000kb	15064
Systolic BP				
BBJ	2.00E-01	0.3	500kb	122815
MVP	1.00E-03	0.1	1000kb	4643
Pan UKBB	1.00E-02	0.1	1000kb	40148
UKBB+ICBP	1.00E-02	0.1	500kb	28613
BBJ+MVP+Pan UKBB	1.00E-04	0.3	250kb	1710
Hypertension				
FINNGEN	1.00E-02	0.3	1000kb	18937
Pan UKBB	2.00E-01	0.3	250kb	302815
FINNGEN+Pan UKBB	1.00E-04	0.1	500kb	2535

For each GWAS, the table describes the characteristics of the selected PRS based on: (1) Genome wide significant SNPs, using SNPs with p-value<$5x10^{-8}$, and fixed clumping parameters: R^2 =0.1 and distance of 1000kb,(2) Optimizing the coefficient of variation across 5 independent subsets of the stage 1 dataset (see Figure 1), and (3) The PRS with the lowest association P-value. We provide clumping criteria (Threshold, R2 and distance) and number of SNP for each GWAS. PRSsum was created by summing the PRS for each criteria.

Supplementary Table 5. Results from association analysis of the number of individuals in longitudinal BP categories and HTN-PRS deciles

**Category**	**beta**	**SE**	**p-value**
Always Hypertension	127.85	10.41	1.80E-06
Worsen	-5.76	4.27	0.21
Improved	-15.08	2.61	4.19E-04
Always Normal & Elevated	-107.07	5.96	9.49E-08

Association analysis was performed studying the association between the number of individuals in a longitudinal BP category and HTN-PRS decile using linear regression.

Supplementary Table 6. Characteristics of the stage 3 CARDIA dataset

	Baseline		Follow up visit I (2 years)		Follow up visit II (5 years)		Follow up visit III (7 years)		Follow up visit IV (10 years)		Follow up visit V (15 years)	
Characteristics	Black	White	Black	White	Black	White	Black	White	Black	White	Black	White
N	1388	1699	1268	1641	1258	1634	1221	1591	1246	1571	1239	1602
Sex (%) F	836 (60.2)	918 (54.0)	765 (60.3)	885 (53.9)	765 (60.2)	878 (54.0)	748 (61.3)	848 (53.3)	757 (60.8)	842 (53.6)	751 (60.6)	865 (54.0)
Age (mean (SD))	24.44 (3.81)	25.67 (3.31)	26.43 (3.80)	27.71 (3.29)	29.51 (3.79)	30.71 (3.30)	31.51 (3.80)	32.68 (3.29)	34.47 (3.80)	35.68 (3.31)	39.49 (3.83)	40.70 (3.30)
BMI (mean (SD))	25.46 (5.80)	23.61 (3.95)	26.40 (6.21)	24.22 (4.22)	27.60 (6.60)	24.86 (4.59)	28.34 (6.77)	25.47 (5.03)	29.14 (7.01)	25.94 (5.25)	30.67 (7.55)	27.18 (5.85)
SBP (mean (SD))	111.43 (11.31)	109.11 (10.93)	109.80 (11.80)	106.45 (10.61)	110.04 (12.44)	105.58 (10.95)	111.48 (13.33)	106.18 (11.53)	113.23 (14.26)	107.18 (11.40)	118.71 (17.98)	110.37 (13.74)
DBP (mean (SD))	68.88 (9.98)	68.40 (9.22)	68.94 (10.41)	66.97 (9.15)	70.98 (11.06)	67.74 (9.55)	71.05 (11.11)	67.89 (9.65)	74.94 (11.15)	70.46 (9.38)	78.06 (13.40)	72.67 (10.58)
Antihypertensive medication (%) Yes	17 (1.2)	11 (0.6)	46 (3.6)	24 (1.5)	35 (2.8)	11 (0.7)	36 (3.0)	17 (1.1)	70 (5.6)	22 (1.4)	163 (13.2)	65 (4.1)
Current Smoke (%) Yes	405 (29.7)	403 (24.1)	494 (39.0)	514 (31.3)	423 (33.6)	436 (26.7)	862 (70.6)	1277 (80.3)	884 (70.9)	1268 (80.7)	895 (72.2)	1328 (82.9)
Hypertension (%) Yes	214 (15.4)	211 (12.4)	207 (16.3)	140 (8.5)	254 (20.2)	175 (10.7)	272 (22.3)	178 (11.2)	369 (29.7)	256 (16.3)	518 (41.9)	389 (24.3)

Supplementary Table 7. Characteristics of the stage 4 MGB Biobank dataset

	African-America	Asian-American	European-American	Hispanic-America	Other	Unknown
N	2039	829	33861	1445	993	1044
Age (mean (SD))	52.85 (16.29)	48.29 (16.23)	60.00 (16.82)	49.95 (16.58)	47.24 (16.24)	53.61 (18.68)
Sex (%) F	1249 (61.3)	483 (58.3)	18050 (53.3)	965 (66.8)	620 (62.4)	532 (51.0)
Hypertension (%)	1068 (52.4)	208 (25.1)	13775 (40.7)	523 (36.2)	298 (30.0)	328 (31.4)
Coronary Artery disease (%)	137 (6.7)	46 (5.5)	2989 (8.8)	68 (4.7)	40 (4.0)	64 (6.1)
Chronic kidney disease (%)	56 (2.7)	9 (1.1)	421 (1.2)	11 (0.8)	7 (0.7)	7 (0.7)
Stroke (%)	33 (1.6)	13 (1.6)	459 (1.4)	20 (1.4)	11 (1.1)	26 (2.5)
Type 2 Diabetes (%)	428 (21.0)	75 (9.0)	2879 (8.5)	257 (17.8)	113 (11.4)	92 (8.8)
Obesity (%)	1112 (54.5)	128 (15.4)	12305 (36.3)	728 (50.4)	456 (45.9)	245 (23.5)

Supplementary figures

Supplementary Figure 1: Association of primary PRS with hypertension in the stage 1 dataset across compared tuning parameter selection criteria

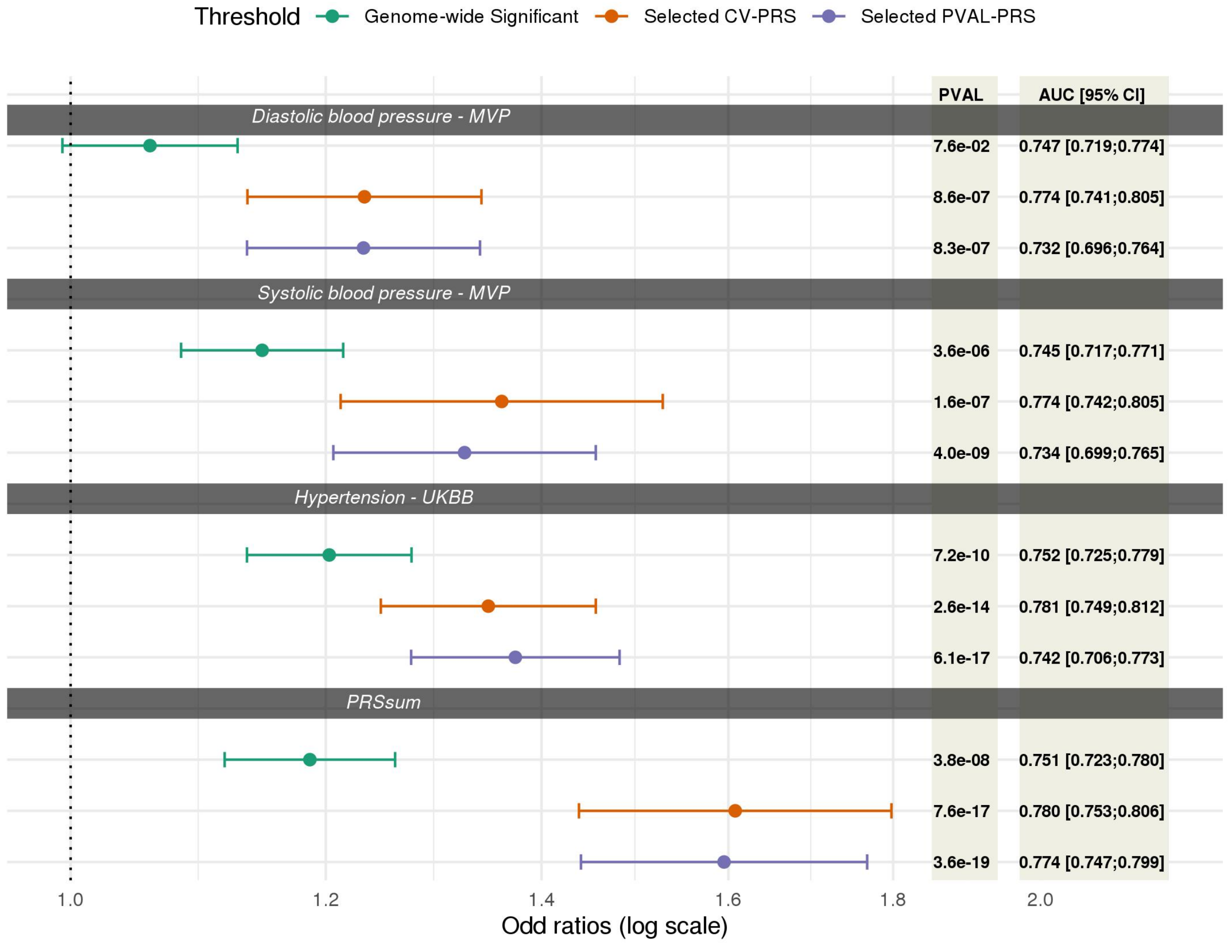

Associations of PRS in stage 1 dataset (N=10,314 individuals). For each GWAS, the PRS were selected based on: (1) “Genome-wide significant PRS” are PRS constructed using genome-wide significant SNPs in the discovery GWAS, with fixed LD parameters or R^2=0.1 and distance =1000kb. (2) “Selected CV PRS” are PRS that minimized the coefficient of variation (CV) across effect size (log odds ratio (OR)) estimates in 5 independent subsets of the Stage 1 dataset. (3)“Selected PVAL-PRS” are PRS that minimized the association p-value with hypertension in the Stage 1 dataset. The figure provides estimated ORs per 1 standard deviation (SD) increase of the PRS, 95% confidence intervals (CIs), p-values from association with hypertension (computed using the Wald test), and Area Under the Receiver Operator Curve (AUC). The PRS association was estimated in a model adjusted for sex, age, age^2, study site, race/ethnic background, smoking status, BMI, and 11 ancestral principal components. PRS SDs were defined according to the sampling SDs of the PRS estimated in the entire TOPMed dataset. Statistical tests relied on the chi-squared distribution with one degree of freedom based on two-sided alternative hypotheses.

Supplementary Figure 2. Association of primary and secondary PRS with hypertension in the BioMe stage 1 dataset across tuning parameter selection criteria

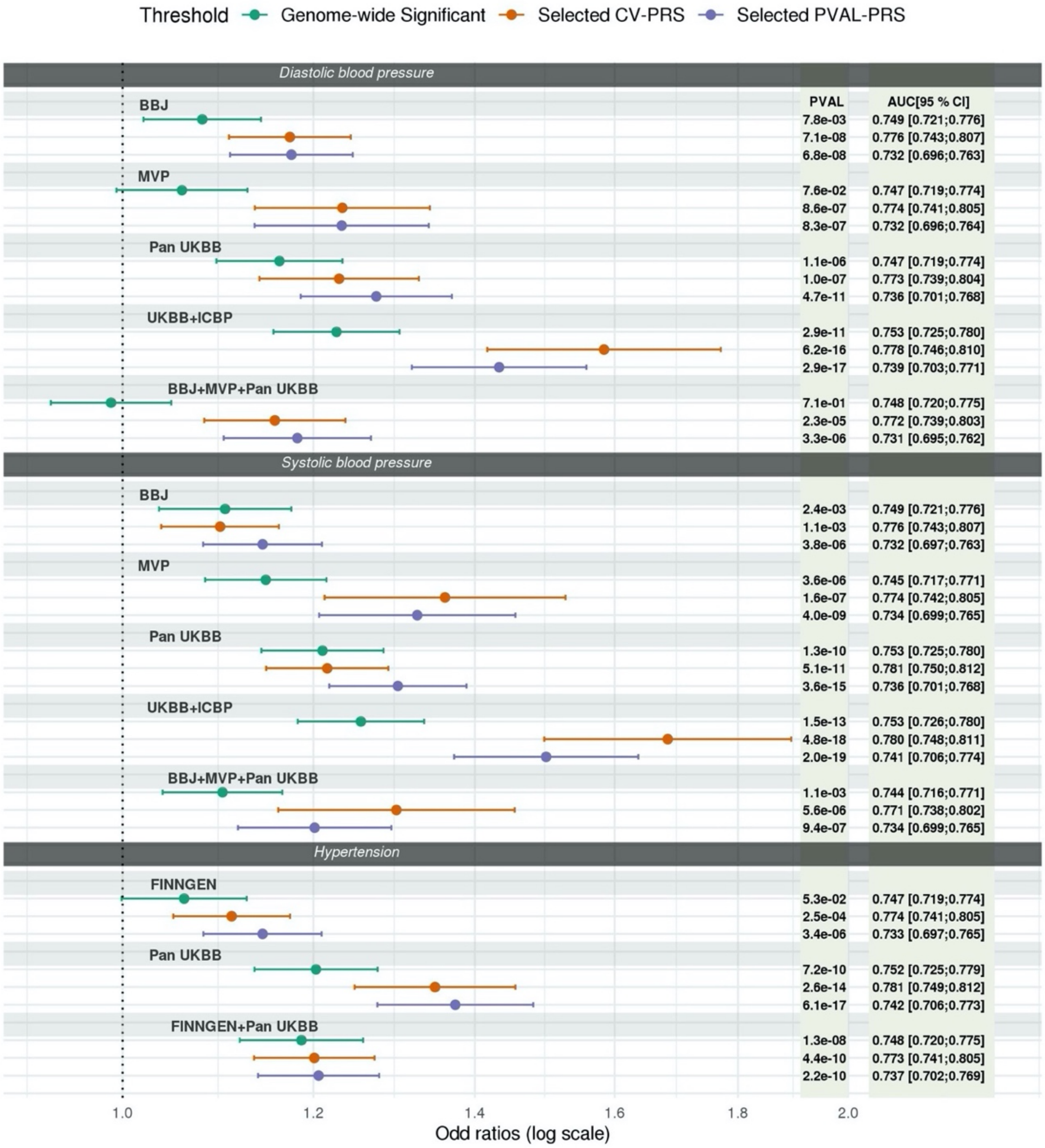

Associations of PRS in stage 1 dataset (N=10,314 individuals). For each of primary and secondary GWAS, the PRS were selected based on: (1) "Genome-wide significant PRS" are PRS constructed using genome-wide significant SNPs in the discovery GWAS, with fixed LD parameters or R^2=0.1 and distance =1000kb. (2) "Selected CV PRS" are PRS that minimized the coefficient of variation (CV) across effect size (log odds ratio (OR)) estimates in 5 independent subsets of the stage 1 dataset. (3)"Selected PVAL-PRS" are PRS that minimized the association p-value with hypertension in the stage 1 dataset. For each tested PRS, the figure provides the estimated OR per 1 standard deviation (SD) increase of the PRS as a point, 95% confidence interval (CI) as an error bar, and in text, its p-value from association analysis with hypertension based on the Wald test, and Area Under the Receiver Operator Curve (AUC). The PRS association was estimated in a model adjusted for sex, age, age^2, study site, race/ethnic background, smoking status, BMI, and 11 ancestral principal components. PRS SDs were defined according to the sampling SDs of the PRS estimated in the entire TOPMed dataset. Statistical tests relied on the chi-squared distribution with one degree of freedom based on two-sided alternative hypotheses.

Supplementary Figure 3. Distribution of longitudinal categories of BP stratified by race/ethnic background

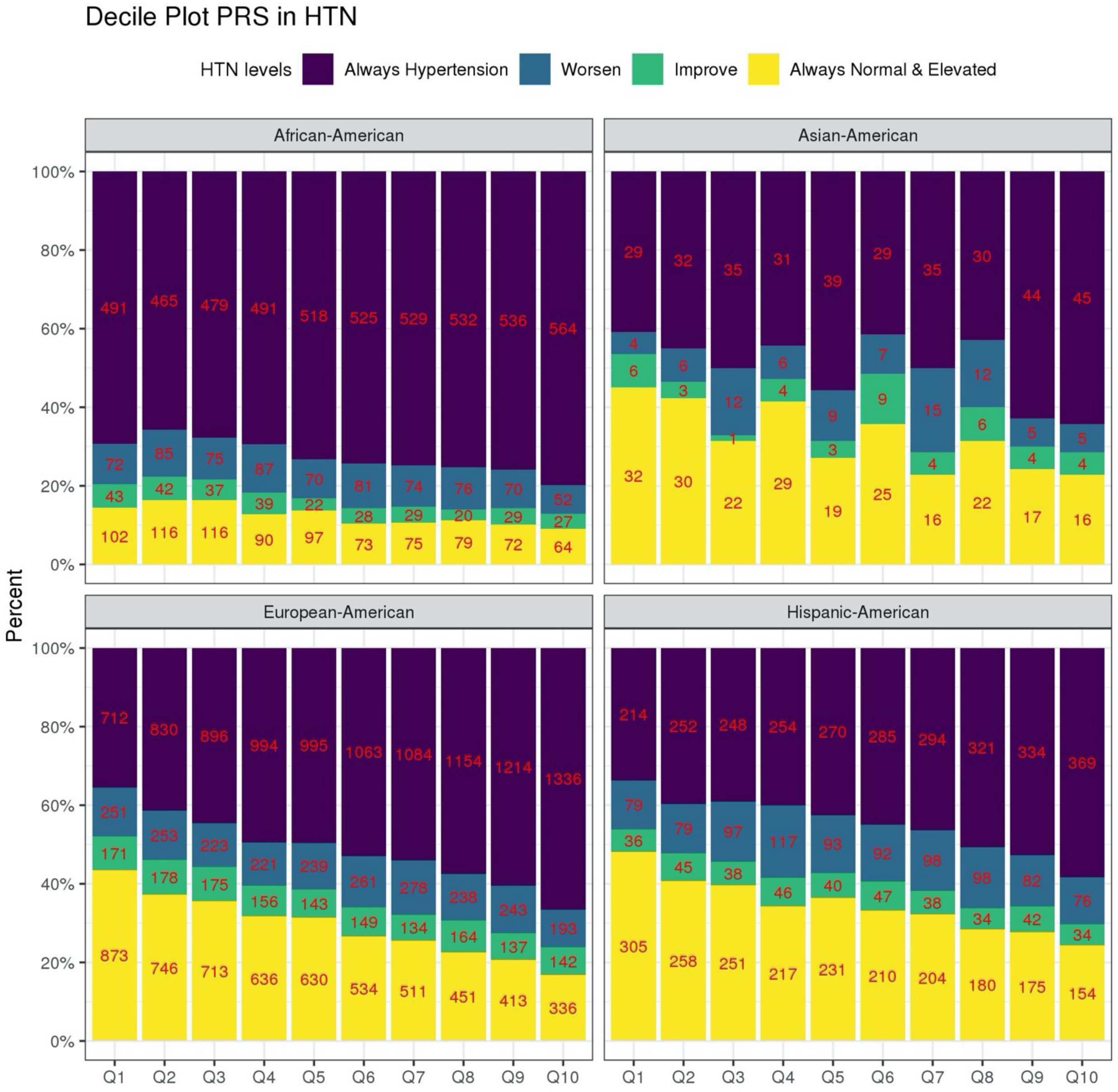

The figure visualizes the distribution of hypertension severity measures stratified by race/ethnic background. Severity measures from most to least severe: always hypertension (treated and hypertension in both visit), worsen (change from normal to elevated BP or hypertension, or change from elevated to hypertension) improve (change from untreated hypertension at to elevated/normal at follow up, or change from elevated to normal), no hypertension in both exams in deciles of the HTN-PRS (PRSsum based on Selected CV-PRS). The numbers provide the sample sizes represented by each bar.

Supplementary Figure 4. Distribution of longitudinal categories of BP stratified by age

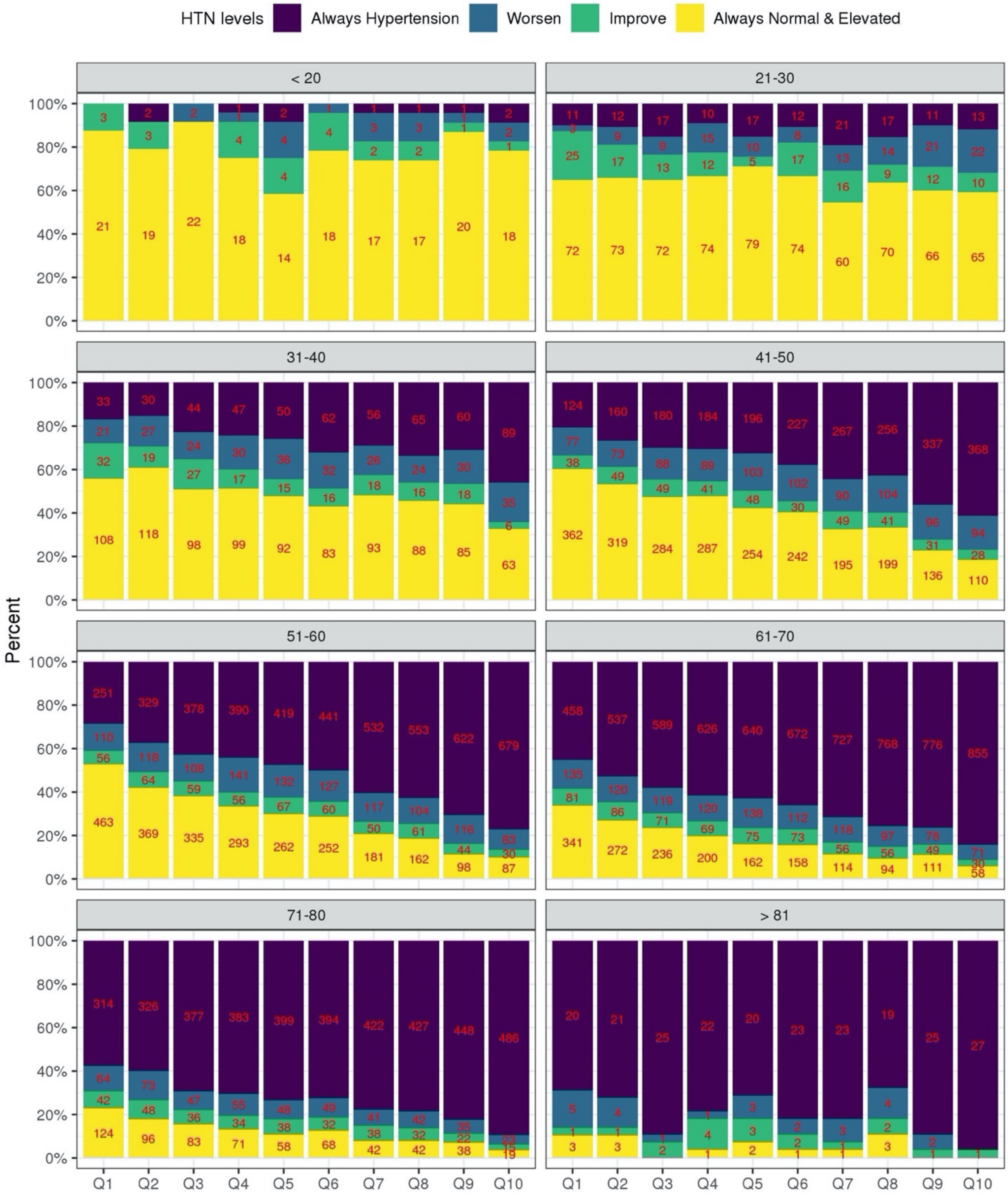

The figure visualizes the distribution of hypertension severity measures stratified by age (age (≤20, 21-30, 31-40, … 71-80, >80). Severity measures from most to least severe: always hypertension (treated and hypertension in both visit), worsen (change from normal to elevated BP or hypertension, or change from elevated to hypertension) improve (change from untreated hypertension at to elevated/normal at follow up, or change from elevated to normal), no hypertension in both exams in deciles of the HTN-PRS (PRSsum based on Selected CV-PRS). The numbers provide the sample sizes represented by each bar.

Supplementary Figure 5: Correlation heatmap between Selected CV PRS by phenotype and PRSsum stratified by race/ethnic background

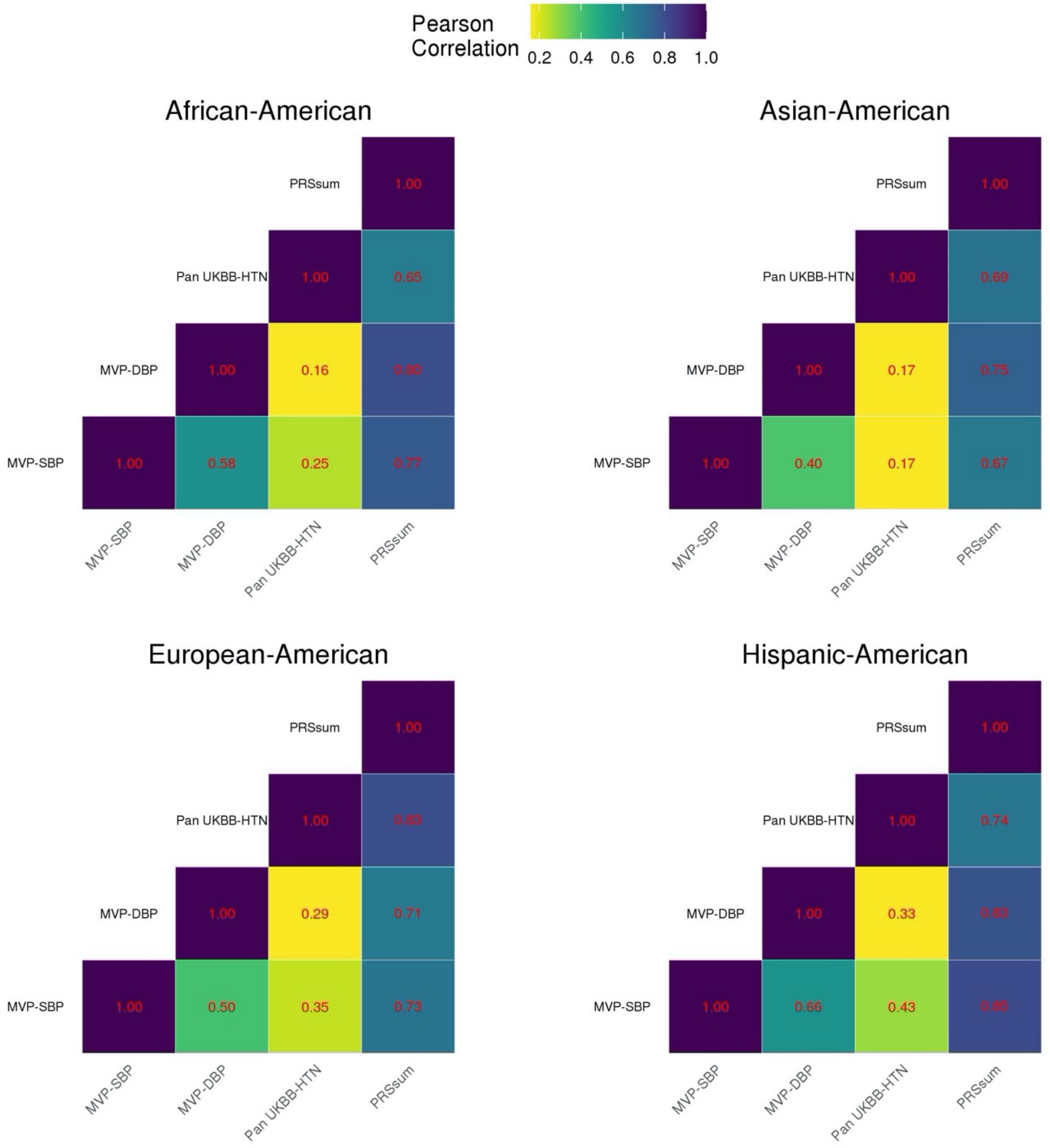

Correlations were computed using the stage 2 dataset.

Supplementary Figure 6: Association of HTN-PRS with hypertension measures across race/ethnicities, with group-specific PRS scaling.

	N	OR [95% CI]	P	AUC
Hypertension at baseline				
African-American	8723	1.32 [1.24;1.41]	3.39e-17	0.753
Asian-American	735	1.34 [1.11;1.62]	2.47e-03	0.562
European-American	22463	1.47 [1.42;1.51]	1.34e-131	0.725
Hispanic-American	6588	1.56 [1.45;1.69]	7.13e-32	0.772
All	37667	2.10 [1.99;2.21]	4.26e-182	0.764
New onset hypertension (normotensive)				
African-American	1131	1.19 [1.02;1.38]	2.86e-02	0.594
Asian-American	260	1.25 [0.85;1.83]	2.61e-01	0.833
European-American	6172	1.32 [1.24;1.41]	3.43e-18	0.563
Hispanic-American	2373	1.38 [1.21;1.58]	2.29e-06	0.757
All	9813	1.72 [1.55;1.91]	4.67e-24	0.656
New onset hypertension (elevated)				
African-American	517	0.93 [0.74;1.18]	5.54e-01	0.812
European-American	2257	1.28 [1.17;1.40]	4.51e-08	0.510
Hispanic-American	758	1.18 [0.96;1.46]	1.13e-01	0.627
All	3531	1.48 [1.27;1.71]	2.39e-07	0.582

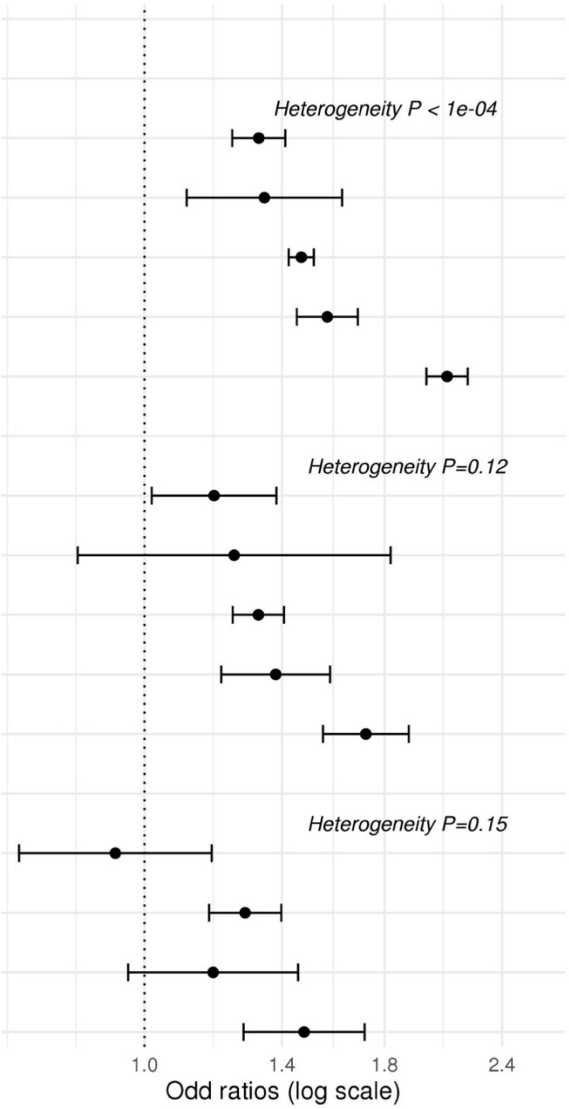

The forest plot provides the association of the HTN-PRS with prevalent and incident hypertension in the stage 2 dataset, and within race/ethnic backgrounds. The top part corresponds to prevalence analysis at the baseline visit, the middle part corresponds to prediction of new onset hypertension in exam 2, among individuals who had normal BP at baseline, and the bottom part corresponds to prediction of new onset hypertension in exam 2, among individuals who had elevated BP at baseline. For each analysis the figure provides sample size (N), estimated odds ratios (ORs) per 1 standard deviation (SD) increase of the PRS, 95% confidence interval (CI), p-value computed using the Wald test, and area under the receiver operating curve (AUC). Estimated ORs and CIs are represented both in text and in points and error bars. Heterogeneity of effects across race/ethnic groups was tested using the Cochran's Q test accounting for correlation due to genetic relatedness across groups. PRS associations was estimated in models adjusted for sex, age, age^2, study site, race/ethnic background, smoking status, BMI, and 11 ancestral principal components. Wald tests relied on the chi-squared distribution with one degree of freedom based on two-sided alternative hypotheses. Cochran's Q tests relied on the chi-squared distribution with degrees of freedom equal to the number of the compared race/ethnic backgrounds minus one.

Supplementary Figure 7. Risk of hypertension in individuals in high deciles of the HTN-PRS compared to those in low deciles of the HTN-PRS.

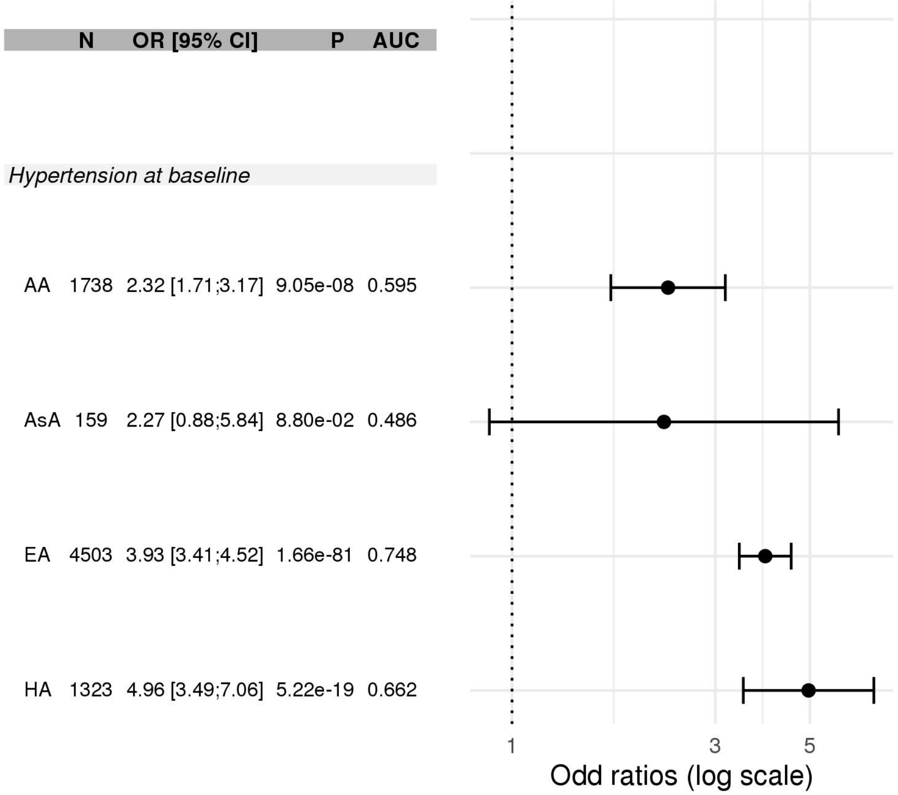

The forest plot provides the association of the HTN-PRS at baseline in individual in the highest versus the lowest decile of the HTN-PRS. Associations were computed in the stage 2 dataset , and are provided in each race/ethnic group separately, because the PRS distribution differ between race/ethnicities. The figure provides sample sizes (N). estimated ORs per 1 standard deviation (SD) increase of the PRSs, 95% confidence intervals (CIs), p-values from association with hypertension based on the Wald test, and Area Under the Receiver Operator Curve (AUC). Estimated ORs and CIs are provided in both text and as points and error bars. The PRS association was estimated in a model adjusted for sex, age, age^2, study site, race/ethnic background, smoking status, BMI, and 11 ancestral principal components. PRS SD was defined according to the sampling SD of the PRS estimated in the entire TOPMed dataset. Statistical tests relied on the chi-squared distribution with one degree of freedom based on two-sided alternative hypotheses.

Supplementary Figure 8. Age-stratified associations of HTN-PRS with prevalent and incident hypertension

	N	OR [95% CI]	P	AUC
Hypertension at baseline				
< 20	236	1.96 [0.72;5.33]	1.86e-01	0.683
21-30	1158	1.14 [0.84;1.55]	3.90e-01	0.671
31-40	2055	1.57 [1.27;1.94]	2.32e-05	0.780
41-50	6616	1.96 [1.76;2.18]	2.46e-33	0.733
51-60	9616	2.11 [1.93;2.32]	1.59e-55	0.711
61-70	11024	2.30 [2.09;2.53]	3.30e-66	0.679
71-80	6276	2.02 [1.76;2.33]	6.44e-23	0.669
> 81	416	2.30 [1.16;4.55]	1.68e-02	0.582
New onset hypertension (normotensive)				
21-30	683	2.44 [1.50;3.99]	3.50e-04	0.568
31-40	1024	1.59 [1.13;2.24]	7.71e-03	0.568
41-50	2716	1.43 [1.17;1.75]	5.52e-04	0.593
51-60	2738	1.82 [1.49;2.23]	5.97e-09	0.626
61-70	1762	2.03 [1.59;2.60]	1.17e-08	0.666
71-80	670	1.64 [1.13;2.37]	9.36e-03	0.515
New onset hypertension (elevated)				
21-30	150	2.21 [0.82;5.95]	1.16e-01	0.481
31-40	215	1.20 [0.60;2.38]	6.09e-01	0.536
41-50	608	1.76 [1.24;2.49]	1.48e-03	0.568
51-60	922	1.15 [0.86;1.54]	3.41e-01	0.646
61-70	1090	1.44 [1.09;1.91]	1.13e-02	0.577
71-80	481	1.80 [1.16;2.78]	8.20e-03	0.611

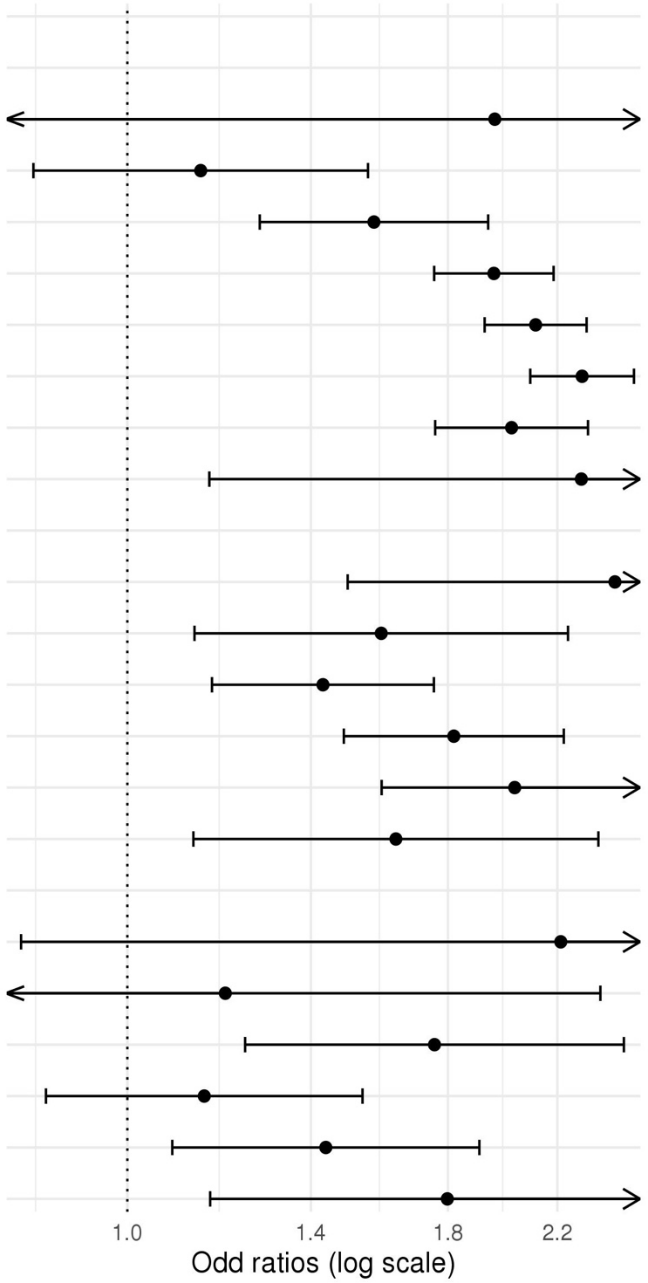

The forest plot provides the association of the HTN-PRS with prevalent and incident hypertension in the stage 2 dataset, and within age groups. Incident hypertension analysis was performed within individuals who had normal BP at baseline (normotensive), and within individuals who had elevated BP at baseline (elevated). For each analysis the figure provide sample size (N), estimated odds ratio (OR) per 1 standard deviation (SD) increase of the PRS , 95% confidence intervals (CIs), p-values from association with hypertension computed using the Wald test, and Area Under the Receiver Operator Curve (AUC). Estimated ORs and CIs are provided both in text and as points with error bars. The PRS associations were estimated in models adjusted for sex, age, age^2, study site, race/ethnic background, smoking status, BMI, and 11 ancestral principal components. PRS SDs were defined according to the sampling SDs of the PRS estimated in the entire TOPMed

dataset. Statistical tests relied on the chi-squared distribution with one degree of freedom based on two-sided alternative hypotheses.

Supplementary Figure 9: Benchmarking the risk of HTN-PRS against other risk factors

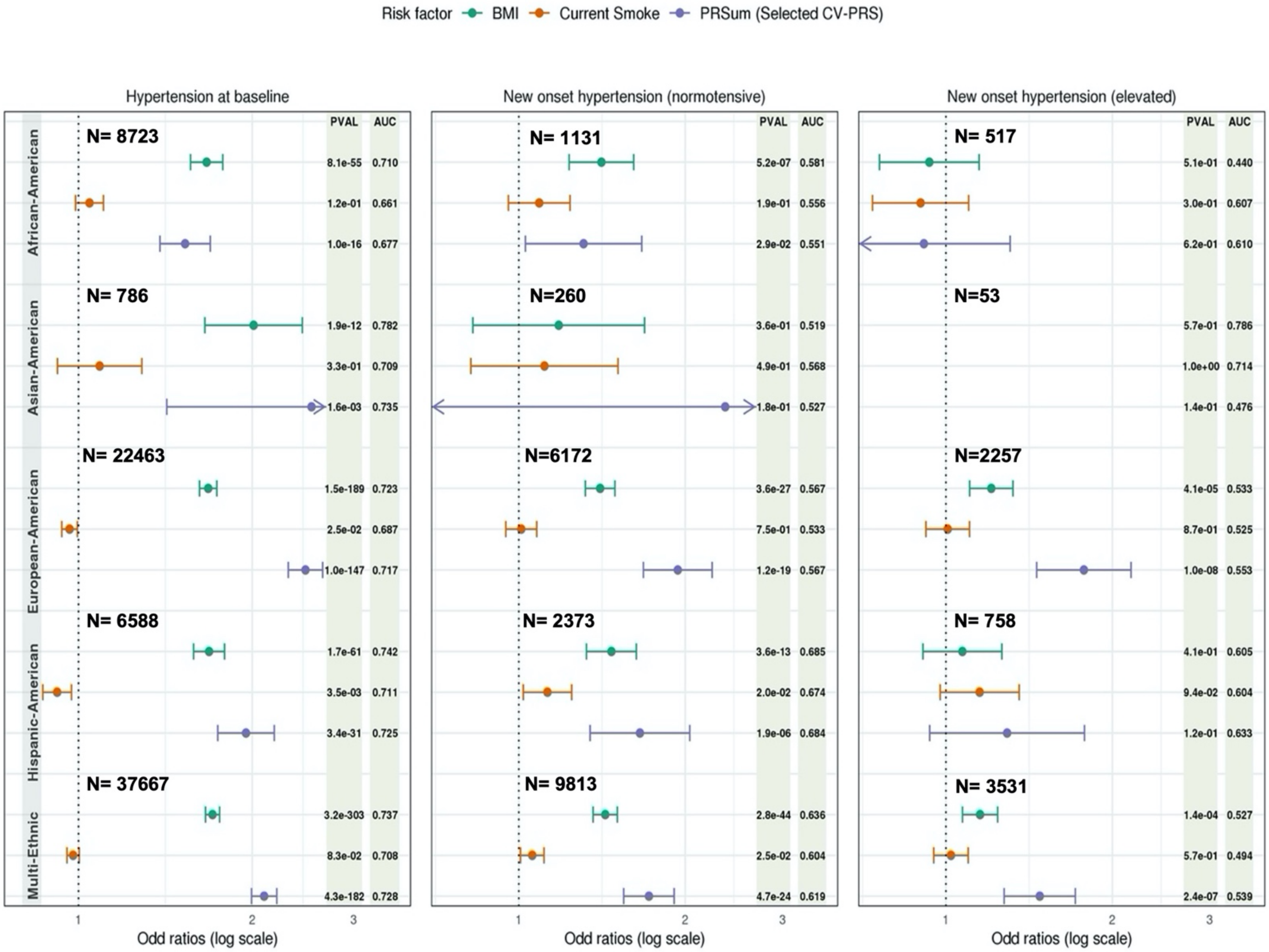

The figure compares hypertension risk factors: BMI, current smoking, and HTN-PRS. For each association analysis, the figure provides the sample size (N), within-group standardized effect size estimate (effect size per 1SD increase in the risk factor within the race/ethnic group used) and 95% confidence interval. These are provided as points and error bars. We also provide sample sizes, p-values from the Wald test, AUCs from a model that include covariates (sex, age, age^2, study site, race/ethnicity in the multi-ethnic model, and 11 PCs) and only the single risk factor of interest (and not the other two risk factors). Statistical tests used the chi-squared test statistic with one degree of freedom based on two-sided alternative hypothesis

Supplementary Figure 10: Race/ethnicity stratified associations of the HTN-PRS with outcomes in the MGB Biobank

	Cases	Controls	OR [95% CI]	P	AUC
Hypertension					
Asian-American	208	621	1.73 [1.22;2.45]	2.23e-03	0.841
African-American	1068	970	1.69 [1.39;2.05]	1.49e-07	0.795
European-American	13773	20081	1.44 [1.39;1.48]	4.14e-115	0.782
Hispanic-American	523	921	1.93 [1.57;2.37]	2.50e-10	0.852
All	16198	24003	1.45 [1.41;1.49]	9.05e-134	0.795
Coronary Artery Disease					
Asian-American	46	783	0.90 [0.47;1.70]	7.40e-01	0.966
African-American	137	1901	1.28 [0.90;1.81]	1.67e-01	0.839
European-American	2989	30865	1.11 [1.05;1.17]	8.86e-05	0.886
Hispanic-American	68	1376	1.89 [1.26;2.83]	1.94e-03	0.911
All	3344	36857	1.13 [1.07;1.18]	3.19e-06	0.894
Type II Diabetes					
Asian-American	75	754	1.71 [1.05;2.77]	2.99e-02	0.783
African-American	428	1610	1.27 [1.02;1.57]	3.36e-02	0.775
European-American	2879	30975	1.18 [1.12;1.24]	1.67e-11	0.731
Hispanic-American	257	1187	1.26 [1.02;1.57]	3.41e-02	0.765
All	3844	36357	1.19 [1.14;1.24]	8.46e-15	0.755
Chronic Kidney Disease					
Asian-American	9	820	2.11 [0.61;7.30]	2.40e-01	0.657
African-American	56	1982	1.41 [0.84;2.36]	1.92e-01	0.648
European-American	421	33433	1.10 [0.98;1.24]	1.11e-01	0.673
Hispanic-American	11	1433	2.71 [1.08;6.81]	3.38e-02	0.764
All	511	39690	1.13 [1.01;1.26]	3.79e-02	0.701
Obesity					
Asian-American	128	701	1.19 [0.85;1.67]	3.16e-01	0.630
African-American	1112	926	0.91 [0.77;1.08]	2.68e-01	0.704
European-American	12301	21553	1.10 [1.07;1.13]	3.88e-12	0.622
Hispanic-American	728	716	0.88 [0.76;1.03]	1.02e-01	0.559
All	14970	25231	1.09 [1.06;1.12]	2.35e-11	0.640
Ischemic Stroke					
Asian-American	13	816	1.75 [0.61;4.98]	2.95e-01	0.891
African-American	33	2005	1.47 [0.76;2.85]	2.49e-01	0.819
European-American	459	33395	1.19 [1.06;1.33]	2.35e-03	0.731
Hispanic-American	20	1424	0.87 [0.45;1.66]	6.70e-01	0.800
All	562	39639	1.15 [1.04;1.28]	6.94e-03	0.757

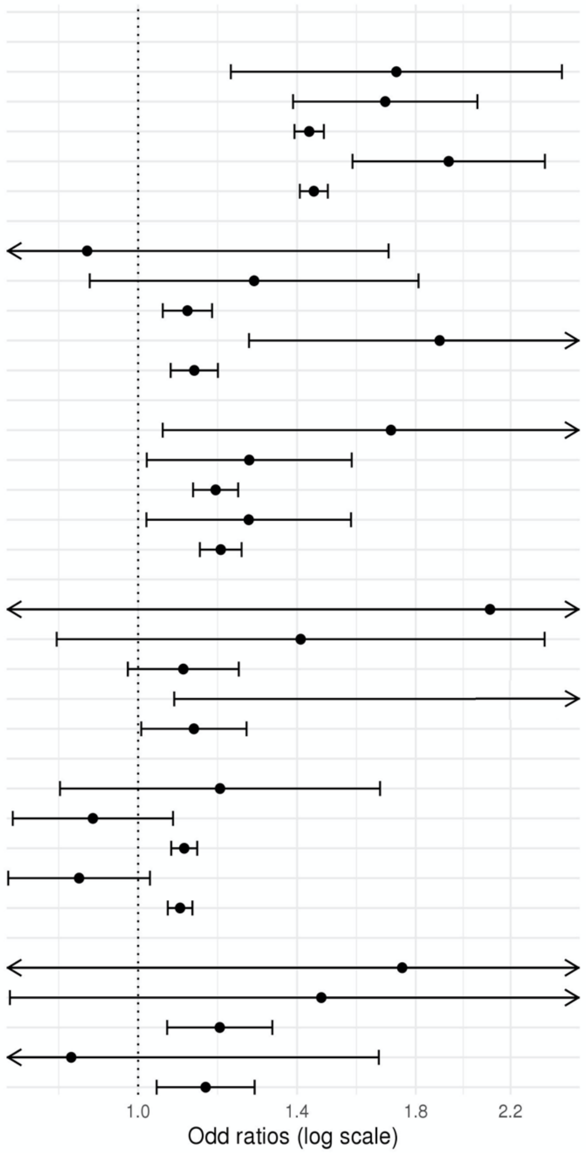

The figure provides the association of HTN-PRS with outcomes in the MGB Biobank, stratified by race/ethnic background groups. For each analysis we provide sample sizes (N cases and N control), estimated odds ratios (ORs) per 1 standard deviation (SD) increase of the PRS, 95% confidence interval (CIs), p-values from association with the outcomes computed using the Wald test, and area under the receiver operating curve (AUC). Estimated ORs and CIs are provided both in text and as points with error bars. PRS associations were estimated in models adjusted for age, sex, race/ethnicity, and 10 ancestral principal components. For scaling PRS, we used pre-compute mean and SD from the complete TOPMed dataset. Statistical tests relied on the chi-squared distribution with one degree of freedom based on two-sided alternative hypotheses.

Supplementary Figure 11: LD-Score regression based estimates of heritability and genetic correlations between BP phentoypes

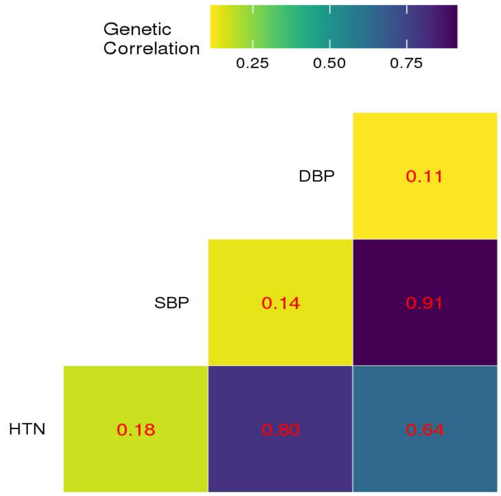

Estimated heritabilities (diagonal) and genetic correlations (off-diagonal) of the BP phenotypes systolic blood pressure (SBP), diastolic blood pressure (DBP) and hypertension (HTN) estimated using LD-Score regression applied on the summary statistics reported in Table 1 in the main manuscript, using our TOPMed dataset as a reference panel for LD.

Supplementary Figure 12: Comparisons of the associations of the HTN-PRS and pleiotropy-based PRS with hypertension in the stage 1 dataset

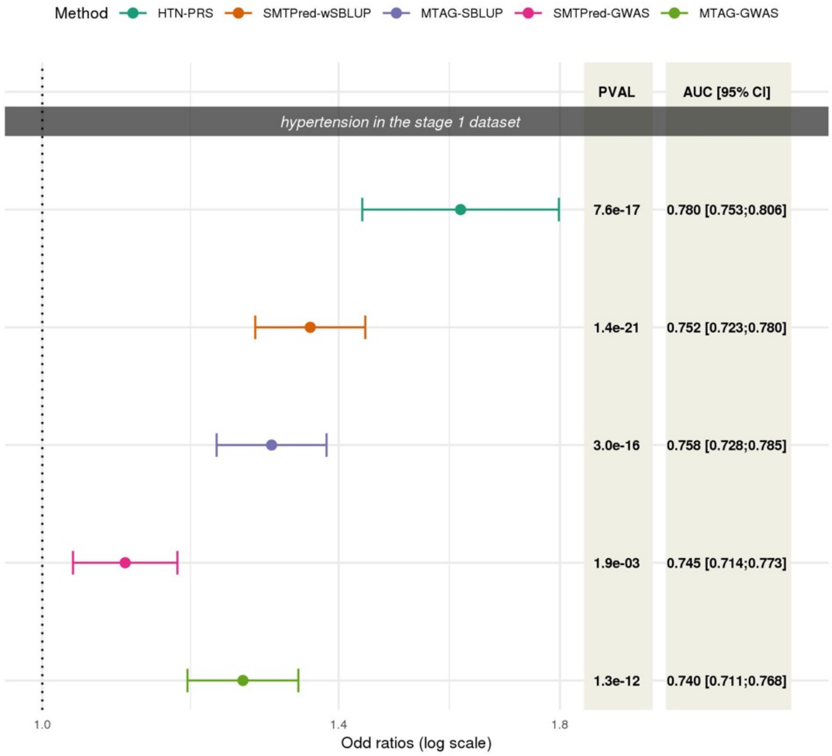

The forest plot provides comparison of the association of the HTN-PRS in the stage 1 dataset (N=10,314 individuals) to other PRS are described in the Supplementary Notes. The other PRS were constructed based on methods that model pleiotropy between the three BP traits. The figure provides estimated ORs per 1 standard deviation (SD) increase of each PRS as points, 95% confidence intervals as error bars, p-values from association with hypertension computed using the Wald test, and Area Under the Receiver Operator Curve (AUC). PRS associations were estimated in models adjusted for sex, age, age^2, study site, race/ethnic background, smoking status, BMI, and 11 ancestral principal components. PRS SDs were defined according to the sampling SDs of the PRS estimated in the entire TOPMed dataset. Statistical tests relied on the chi-squared distribution with one degree of freedom based on two-sided alternative hypotheses.

Supplementary Figure 13: Comparisons of the associations of the HTN-PRS and pleiotropy-based PRS with hypertension in the stage 2 dataset

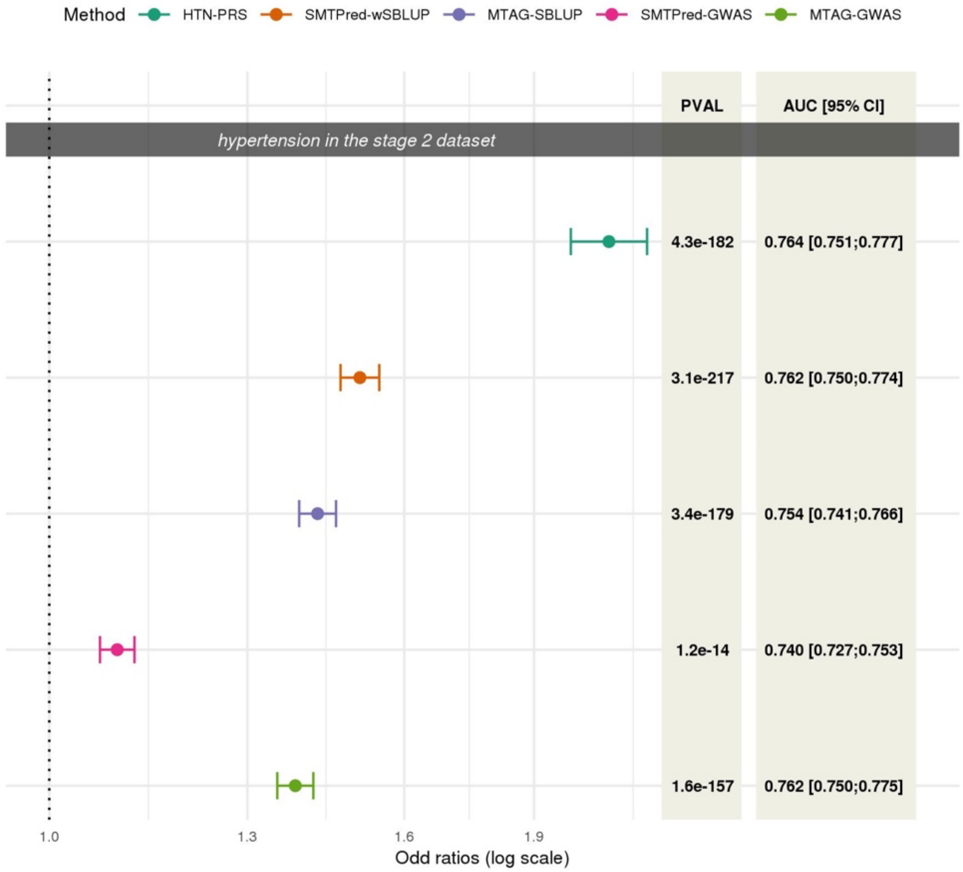

Associations of the HTN-PRS and pleiotropy-based PRS described in the Supplementary Notes in the stage 2 dataset (N =37,667 individuals). The figure provides estimated ORs per 1 standard deviation (SD) increase of each PRS represented as points, 95% confidence intervals represented as error bars, p-values from association with hypertension based on the Wald test, and Area Under the Receiver Operator Curve (AUC). PRS associations were estimated in models adjusted for sex, age, age^2, study site, race/ethnic background, smoking status, BMI, and 11 ancestral principal components. PRS SD was defined according to the sampling SD of the PRS estimated in the entire TOPMed dataset. Statistical tests relied on the chi-squared distribution with one degree of freedom based on two-sided alternative hypotheses.

Supplementary Figure 14: BP trait PRS distribution stratified by groups defined by genetic ancestry

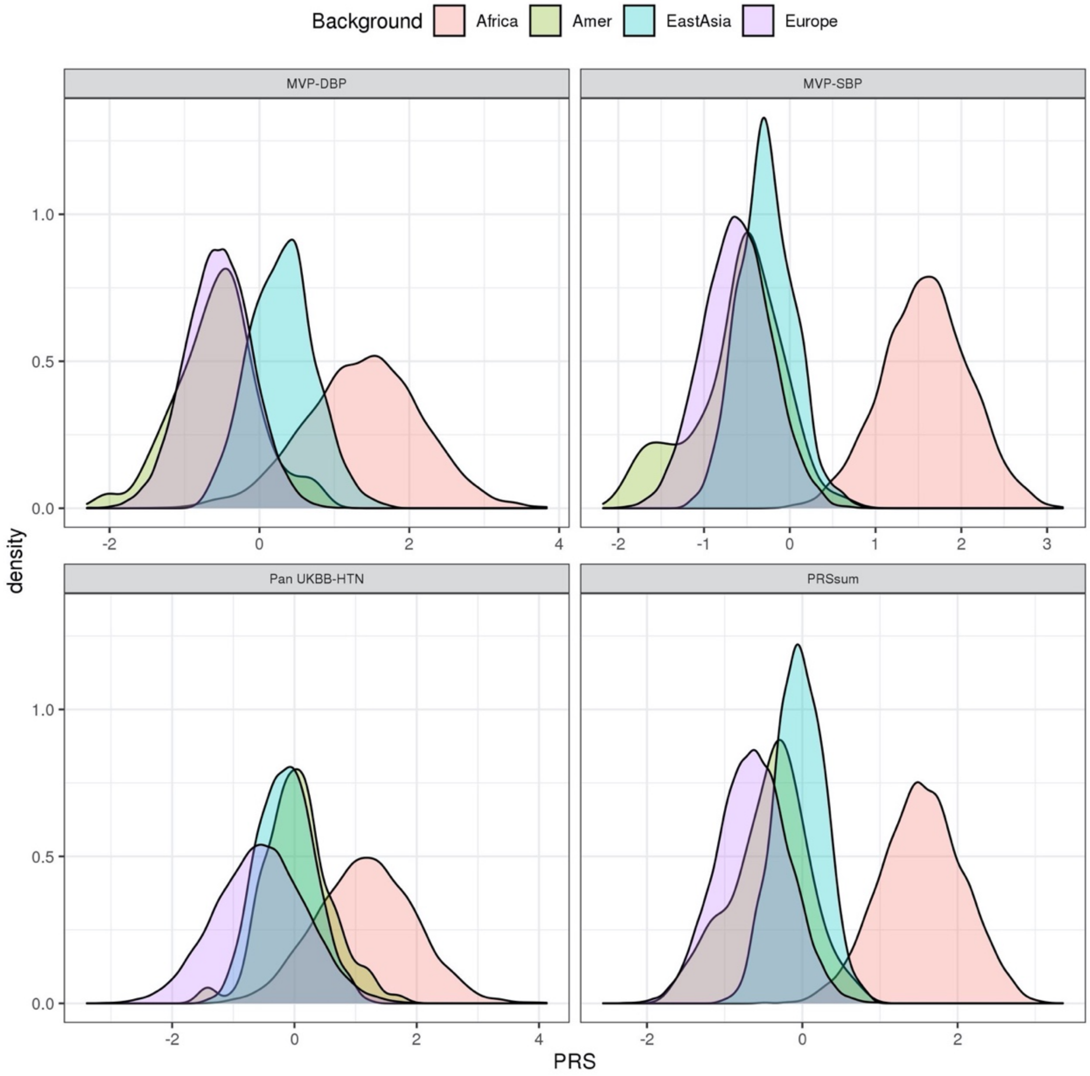

Density plots showing the distributions of Selected CV-PRS based on each GWAS used (Table 1 in the main manuscript) and PRSsum constructed by summing Selected CV-PRS from the three GWAS (the final HTN-PRS). The figure was created using the stage 2 dataset. The densities are stratified by groups composed by individuals having at least 80% of their genomes from either African, Amerindian (Amer), East Asian, or European genetic ancestry.

Supplementary Figure 15: Association of HTN-PRS with hypertension measures across groups defined by high proportions of genetic ancestries

	N	OR [95% CI]	P	AUC
Hypertension at baseline				
All	37667	2.10 [1.99; 2.21]	4.26e-182	0.764
Africa	5447	1.54 [1.35; 1.77]	2.78e-10	0.760
Amer	94	1.45 [0.10;21.15]	7.85e-01	0.750
East Asia	783	2.41 [1.36; 4.28]	2.69e-03	0.547
Europe	20939	2.34 [2.19; 2.51]	8.18e-128	0.753
New onset hypertension (normotensive)				
All	9813	1.72 [1.55; 1.91]	4.67e-24	0.656
Africa	677	1.24 [0.91; 1.71]	1.74e-01	0.588
East Asia	259	2.62 [0.80; 8.57]	1.10e-01	0.500
Europe	5748	1.94 [1.68; 2.24]	2.94e-19	0.657
New onset hypertension (elevated)				
All	3531	1.48 [1.27; 1.71]	2.39e-07	0.582
Africa	321	0.91 [0.54; 1.55]	7.33e-01	0.517
Europe	2127	1.73 [1.42; 2.12]	6.15e-08	0.573

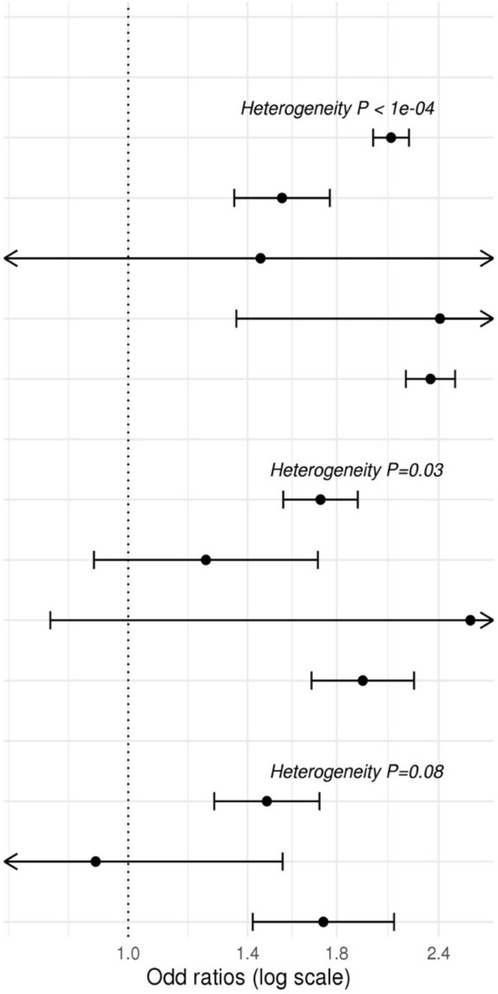

The forest plot provides the association of the HTN- PRS with prevalent and incident hypertension in the stage 2 dataset, and within groups composed by individuals having at least 80% of their genomes from either African, Amerindian (Amer), East Asian, or European genetic ancestry. The top part corresponds to prevalence analysis at the baseline visit, the middle part corresponds to prediction of new onset hypertension in exam 2, among individuals who had normal BP at baseline, and the bottom part corresponds to prediction of new onset hypertension in exam 2, among individuals who had elevated BP at baseline. For each analysis the figure provides sample size (N), estimated odds ratios (OR) per 1 standard deviation (SD) increase of the PRS, and 95% confidence interval (CI), p-value computed using the Wald test, and area under the receiver operating curve (AUC). Estimated ORs and CIs are provided both as text and as points with error bars. Heterogeneity of effects across race/ethnic groups was tested using the Cochran's Q test accounting for correlation due to genetic relatedness across groups. PRS associations were estimated in models adjusted for sex, age, age^2, study site, race/ethnic background, smoking status, BMI, and 11 ancestral principal components. PRS SD was defined according to the sampling SD of the PRS estimated in the entire TOPMed dataset. Wald tests relied on the chi-squared distribution with one degree of freedom based on two-sided alternative hypotheses. Cochran's Q tests relied on the chi-squared distribution with degrees of freedom equal to the number of the compared race/ethnic backgrounds minus one.

Supplementary Note 3: Study descriptions

BioMe

The BioMe Biobank is an ongoing, prospective, hospital- and outpatient- based population research program operated by The Charles Bronfman Institute for Personalized Medicine (IPM) at Mount Sinai. BioMe has enrolled over 50,000 participants between September 2007 and July 2019. BioMe is an Electronic Medical Record (EMR)-linked biobank that integrates research data and clinical care information for consented patients at The Mount Sinai Medical Center, which serves diverse local communities of upper Manhattan with broad health disparities. IPM BioMe populations include 25% of African American ancestry (AA), 36% of Hispanic Latino ancestry (HL), 30% of white European ancestry (EA), and 9% of other ancestry. The BioMe disease burden is reflective of health disparities in the local communities. BioMe operations are fully integrated in clinical care processes, including direct recruitment from clinical sites waiting areas and phlebotomy stations by dedicated BioMe recruiters independent of clinical care providers, prior to or following a clinician standard of care visit. Recruitment currently occurs at a broad spectrum of over 30 clinical care sites.

Blood pressure measurements methods:

BioMe operations are fully integrated in clinical care processes, including direct recruitment from clinical sites waiting areas and phlebotomy stations by dedicated BioMe recruiters independent of clinical care providers, prior to or following a clinician standard of care visit. Recruitment currently occurs at a broad spectrum of over 30 clinical care sites. Information on anthropometrics, demographics, blood pressure values and use of antihypertensive medication

was derived from participants Electronic Medical Record (EMR).

Ethics statement:

The BioMe cohort was approved by the Institutional Review Board at the Icahn School of Medicine at Mount Sinai. All BioMe participants provided written, informed consent for genomic data sharing.

BioMe acknowledgements:

The Mount Sinai BioMe Biobank has been supported by The Andrea and Charles Bronfman Philanthropies and in part by Federal funds from the NHLBI and NHGRI (U01HG00638001; U01HG007417; X01HL134588). We thank all participants in the Mount Sinai Biobank. We also thank all our recruiters who have assisted and continue to assist in data collection and management and are grateful for the computational resources and staff expertise provided by Scientific Computing at the Icahn School of Medicine at Mount Sinai.

ARIC

The Atherosclerosis Risk in Communities (ARIC) study (dbGaP accession phs000090) is a population-based prospective cohort study of cardiovascular disease sponsored by the NHLBI. ARIC included 15,792 individuals, predominantly European American and African American, aged 45-64 years at baseline (1987-89), chosen by probability sampling from four US communities. Cohort members completed three additional triennial follow-up examinations, a fifth exam in 2011-2013, a sixth exam in 2016-2017, and a seventh exam in 2018-2019. The

ARIC study has been described in detail previously [9]. For this analysis, we used BP measurements from the first and third ARIC exams.

Blood pressure measurements methods:

BP was measured using a standardized Hawksley random-zero mercury column sphygmomanometer with participants in a sitting position after a resting period of 5 minutes. The size of the cuff was chosen according to the arm circumference. Three sequential recordings for SBP and DBP were obtained; the mean of the last two measurements was used in this analysis, discarding the first reading. Blood pressure lowering medication use was recorded from the medication history

Ethics statement:

The ARIC study has been approved by Institutional Review Boards (IRB) at all participating institutions: University of North Carolina at Chapel Hill IRB, Johns Hopkins University IRB, University of Minnesota IRB, and University of Mississippi Medical Center IRB. Study participants provided written informed consent at all study visits.

ARIC acknowledgements:

The Atherosclerosis Risk in Communities study has been funded in whole or in part with Federal funds from the National Heart, Lung, and Blood Institute, National Institutes of Health, Department of Health and Human Services (contract numbers HHSN268201700001I,

HHSN268201700002I, HHSN268201700003I, HHSN268201700004I and HHSN268201700005I).
The authors thank the staff and participants of the ARIC study for their important contributions.

CHS

The Cardiovascular Health Study (CHS) is a population-based cohort study initiated by the National Heart, Lung and Blood Institute (NHLBI) in 1987 to determine the risk factors for development and progression of cardiovascular disease (CVD) in older adults, with an emphasis on subclinical measures. The study recruited 5,888 adults aged 65 or older at entry in four U.S. communities and conducted extensive annual clinical exams between 1989-1999 along with semi-annual phone calls, events adjudication, and subsequent data analyses and publications [10]. Additional data are collected by studies ancillary to CHS. In June 1990, four Field Centers (Sacramento, CA; Hagerstown, MD; Winston-Salem, NC; Pittsburgh, PA) completed the recruitment of 5201 participants. Between November 1992 and June 1993, an additional 687 participants, primarily African American, were recruited using similar methods. Blood samples were drawn from all participants at their baseline examination and during follow-up clinic visits, and DNA was subsequently extracted from available samples. CHS analyses were limited to participants with available DNA who consented to genetic studies. The baseline examinations consisted of a home interview and a clinic examination that assessed not only traditional risk factors but also measures of subclinical disease, including carotid ultrasound, echocardiography, electrocardiography, and pulmonary function. Between enrollment and 1998-99, participants were seen in the clinic annually and contacted by phone at 6-month intervals to collect information about hospitalizations and potential cardiovascular events.

Major exam components were repeated during annual follow-up examinations through 1999. Standard protocols for the identification and adjudication of events were implemented during follow-up. The adjudicated events are CHD, angina, heart failure (HF), stroke, transient ischemic attack (TIA), claudication and mortality. Adjudication of cause of death continues using a streamlined protocol; adjudication of other events ended in June 2015. Since 1999, participants have been contacted every 6 months by phone, primarily to ascertain health status and for events follow-up. The study was initially approved by institutional review boards at the Field Centers (Wake Forest, University of California – Davis, Johns Hopkins University, University of Pittsburgh), the Core Laboratory (University of Vermont) and at the Coordinating Center (University of Washington). The University of Washington now handles CHS Data Repository approvals.

Blood pressure measurements methods:

Research staff who received central training in blood pressure measurement assessed repeat right-arm seated systolic and diastolic blood pressure levels at baseline with a Hawksley random-zero sphygmomanometer. Means of the repeated blood pressure measurements from the baseline examination were used for these analyses. Blood pressure lowering medication use was recorded from the medication history.

Ethics statement:

All CHS participants provided informed consent, and the study was approved by the Institutional Review Board [or ethics review committee] of University Washington.

CHS acknowledgements:

Cardiovascular Health Study: This research was supported by contracts HHSN268201200036C, HHSN268200800007C, HHSN268201800001C, N01HC55222, N01HC85079, N01HC85080, N01HC85081, N01HC85082, N01HC85083, N01HC85086, 75N92021D00006, and grants U01HL080295, U01HL130114, and HL105756 from the National Heart, Lung, and Blood Institute (NHLBI), with additional contribution from the National Institute of Neurological Disorders and Stroke (NINDS). Additional support was provided by R01AG023629 from the National Institute on Aging (NIA). A full list of principal CHS investigators and institutions can be found at CHS-NHLBI.org. The content is solely the responsibility of the authors and does not necessarily represent the official views of the National Institutes of Health.

CARDIA

The Coronary Artery Risk Development in Young Adults study (dbGaP accession phs000285) is a prospective multicenter study with 5,115 adults Caucasian and African American participants of the age group 18-30 years at baseline, recruited from four centers at the baseline examination in 1985-1986 [11]. The recruitment was done from the total community in Birmingham, AL, from selected census tracts in Chicago, IL and Minneapolis, MN; and from the Kaiser Permanente health plan membership in Oakland, CA. Nine examinations have been completed in the years 0, 2, 5, 7, 10, 15, 20, 25 and 30, with high retention rates (91%, 86%, 81%, 79%, 74%, 72%, 72%, and 71%, respectively) and written informed consent was obtained in each visit.

Blood pressure measurements methods:

Seated BP was measured on the right arm following 5 minutes rest using a random-zero sphygmomanometer. SBP and DBP were recorded as Phase I and Phase V Korotkoff sounds. Three measurements were taken at 1 minute intervals with the average of the second and third measurements taken for the BP values.

Ethics statement:

All CARDIA participants provided informed consent, and the study was approved by the Institutional Review Boards of the University of Alabama at Birmingham and the University of Texas Health Science Center at Houston.

CARDIA acknowledgements:

The Coronary Artery Risk Development in Young Adults Study (CARDIA) is conducted and supported by the National Heart, Lung, and Blood Institute (NHLBI) in collaboration with the University of Alabama at Birmingham (HHSN268201800005I & HHSN268201800007I), Northwestern University (HHSN268201800003I), University of Minnesota (HHSN268201800006I), and Kaiser Foundation Research Institute (HHSN268201800004I). This manuscript has been reviewed by CARDIA for scientific content.

FHS

The Framingham Heart Study (dbGaP accession phs000007) began in 1948 with the recruitment of an original cohort of 5,209 men and women (mean age 44 years; 55 percent women). In 1971 a second generation of study participants was enrolled; this cohort (mean age 37 years;

52% women) consisted of 5,124 children and spouses of children of the original cohort. A third-generation cohort of 4,095 children of offspring cohort participants (mean age 40 years; 53 percent women) was enrolled in 2002-2005 and are seen every 4 to 8 years. Details of study designs for the three cohorts are summarized elsewhere [12–14]. At each clinic visit, a medical history was obtained, and participants underwent a physical examination. Only study participants consented for genetic and non-genetic data are included. FHS has been approved by the Boston University IRB

Blood pressure measurements methods:

Systolic and diastolic blood pressures were measured twice by a physician on the left arm of the resting and seated participants using a mercury column sphygmomanometer. The blood pressure measurements used in this study were obtained at examination one cycle for all three generation cohorts.

Ethics statement:

The Framingham Heart Study was approved by the Institutional Review Board of the Boston University Medical Center. All study participants provided written informed consent.

FHS acknowledgements:

The Framingham Heart Study (FHS) acknowledges the support of contracts NO1-HC-25195, HHSN268201500001I and 75N92019D00031 from the National Heart, Lung and Blood Institute

and grant supplement R01 HL092577-06S1 for this research. We also acknowledge the dedication of the FHS study participants without whom this research would not be possible.

GENOA

The Genetic Epidemiology Network of Arteriopathy (GENOA) study, a part of the Family Blood Pressure Program (FBPP Investigators, 2002), consists of hypertensive sibships that were recruited for linkage and association studies in order to identify genes that influence blood pressure and its target organ damage (Daniels, 2004). In the initial phase of the GENOA study (Phase I: 1996-2001), all members of sibships containing ≥ 2 individuals with essential hypertension clinically diagnosed before age 60 were invited to participate, including both hypertensive and normotensive siblings. In the second phase of the GENOA study (Phase II: 2000-2004), 1,239 non-Hispanic white and 1,482 African American participants were successfully re-recruited to measure potential target organ damage due to hypertension.

Blood pressure measurements methods:

SBP and DBPs were measured using an automated oscillometric BP measurement device with a consistent protocol across the FBPP networks. BP was measured three times on each participant by trained and certified technicians and then averaged for use in this analysis.

Ethics statement:

Written informed consent was obtained from all subjects and approval was granted by participating institutional review boards (University of Michigan, University of Mississippi Medical Center, and Mayo Clinic).

GENOA acknowledgements:

Support for the Genetic Epidemiology Network of Arteriopathy (GENOA) was provided by the National Heart, Lung and Blood Institute (U01 HL054457, U01 HL054464, U01 HL054481, R01 HL119443, and R01 HL087660) of the National Institutes of Health. We would like to thank the GENOA participants.

HCHS/SOL

The Hispanic Community Health Study/Study of Latinos (dbGaP accession phs000810) is a community-based longitudinal cohort study of 16,415 self-identified Hispanic/Latino persons aged 18–74 years and selected from households in predefined census-block groups across four US field centers (in Chicago, Miami, the Bronx, and San Diego). The census-block groups were chosen to provide diversity among cohort participants with regard to socioeconomic status and national origin or background [15,16]. The HCHS/SOL cohort includes participants who self-identified as having a Hispanic/Latino background; the largest groups are Central American (n = 1,730), Cuban (n = 2,348), Dominican (n = 1,460), Mexican (n = 6,471), Puerto Rican (n = 2,728), and South American (n = 1,068). The HCHS/SOL baseline clinical examination occurred between 2008 and 2011 and included comprehensive biological, behavioral, and sociodemographic assessments. Visit 2 took place between 2014 and 2017, which re-examined 11,623 participants

from the baseline sample. Visit 3 has started in 2020 and will last 3 years. In addition to clinic visit, participants are contacted annually to assess clinical outcomes. The study was approved by the Institutional Review Boards at each participating institution and written informed consent was obtained from all participants.

Blood pressure measurements methods:

Blood pressure was measured on the right arm using an OMRON HEM-907 XL (Omron Healthcare, Inc., Lake Forest, IL) automatic sphygmomanometer, with the participant in the seated position and the arm resting. Cuff sizes were determined by measurement of the upper arm circumference. Four cuff sizes were available. Three blood pressures measurements were obtained 1 minute apart following an initial 5-minute rest period. The average of these 3 blood pressure values was used in this analysis. If there were fewer than 3 measurements, all available measurements were averaged. For more details, see [17].

Ethics statement:

This study was approved by the institutional review boards (IRBs) at each field center, where all participants gave written informed consent, and by the Non-Biomedical IRB at the University of North Carolina at Chapel Hill, to the HCHS/SOL Data Coordinating Center. All IRBs approving the study are: Non-Biomedical IRB at the University of North Carolina at Chapel Hill. Chapel Hill, NC; Einstein IRB at the Albert Einstein College of Medicine of Yeshiva University. Bronx, NY; IRB at Office for the Protection of Research Subjects (OPRS), University of Illinois at Chicago. Chicago,

IL; Human Subject Research Office, University of Miami. Miami, FL; Institutional Review Board of San Diego State University. San Diego, CA.

HCHS/SOL acknowledgements:

The Hispanic Community Health Study/Study of Latinos is a collaborative study supported by contracts from the National Heart, Lung, and Blood Institute (NHLBI) to the University of North Carolina (HHSN268201300001I / N01-HC-65233), University of Miami (HHSN268201300004I / N01-HC- 65234), Albert Einstein College of Medicine (HHSN268201300002I / N01-HC-65235), University of Illinois at Chicago – HHSN268201300003I / N01- HC-65236 Northwestern Univ), and San Diego State University (HHSN268201300005I / N01-HC-65237). The following Institutes/Centers/Offices have contributed to the HCHS/SOL through a transfer of funds to the NHLBI: National Institute on Minority Health and Health Disparities, National Institute on Deafness and Other Communication Disorders, National Institute of Dental and Craniofacial Research, National Institute of Diabetes and Digestive and Kidney Diseases, National Institute of Neurological Disorders and Stroke, NIH Institution-Office of Dietary Supplements.

JHS

The Jackson Heart Study (dbGaP accession phs000286) is a longitudinal investigation of genetic and environmental risk factors associated with the disproportionate burden of cardiovascular disease in African Americans [18,19]. JHS is funded by the NHLBI and the National Institute on Minority Health and Health Disparities (NIMHD). JHS is an expansion of the ARIC study in its Jackson Field Center. At baseline, the JHS recruited 5306 African American residents of the

Jackson Mississippi Metropolitan Statistical Area aged, approximately 6.6% of all African American adults aged 35-84 residing in the area. Participants were recruited via random sampling (17% of participants), volunteers (30%), prior participants in the Atherosclerosis Risk in Communities (ARIC) study (31%), and secondary family members (22%). Among these participants, approximately 3400 gave consent that allows genetic research. JHS participants received three back-to-back clinical examinations (Exam 1, 2000-2004; Exam 2, 2005-2008; and Exam 3, 2009-2013), and a fourth clinical examination has started in 2020. Participants are also contacted annually by telephone to update personal and health information including vital status, interim medical events, hospitalizations, functional status and sociocultural information

Blood pressure measurements methods:

Two seated blood pressure measurements were taken using a Hawksley random zero sphygmomanometer and an appropriately sized cuff. BP measurements were calibrated using robust regression to the Omron HEM-907XL device [20]. The use of antihypertensive medications was recorded in the medication history.

Ethics statement:

The JHS study was approved by Jackson State University, Tougaloo College, and the University of Mississippi Medical Center IRBs, and all participants provided written informed consent.

JHS acknowledgements:

The Jackson Heart Study (JHS) is supported and conducted in collaboration with Jackson State University (HHSN268201800013I), Tougaloo College (HHSN268201800014I), the Mississippi State Department of Health (HHSN268201800015I) and the University of Mississippi Medical Center (HHSN268201800010I, HHSN268201800011I and HHSN268201800012I) contracts from the National Heart, Lung, and Blood Institute (NHLBI) and the National Institute for Minority Health and Health Disparities (NIMHD). The authors also wish to thank the staffs and participants of the JHS.

WHI

The Women's Health Initiative (WHI) cohort. The WHI is a prospective national health study focused on identifying optimal strategies for preventing chronic diseases that are the major causes of death and disability in postmenopausal women [21]. The WHI initially recruited 161,808 women between 1993 and 1997 with the goal of including a socio-demographically diverse population with racial/ethnic minority groups proportionate to the total minority population of US women aged 50-79 years. The WHI consists of two major parts: a set of randomized Clinical Trials and an Observational Study. The WHI Clinical Trials (CT; N=68,132) includes three overlapping components, each a randomized controlled comparison: the Hormone Therapy Trials (HT), Dietary Modification Trial, and Calcium and Vitamin D Trial. A parallel prospective observational study (OS; N = 93,676) examined biomarkers and risk factors associated with various chronic diseases. While the HT trials ended in the mid-2000s, active follow-up of the WHI-CT and WHI-OS cohorts has continued for over 25 years, with the accumulation of large

numbers of diverse clinical outcomes, risk factor measurements, medication use, and many other types of data.

Blood pressure measurements methods:

BP was measured by certified staff using standardized procedures and instruments[22]. Two BP measures were recorded after 5 minutes rest using a mercury sphygmomanometer. Appropriate cuff bladder size was determined at each visit based on arm circumference. Diastolic BP was taken from the phase V Korotkoff measures. The average of the two measurements, obtained 30 seconds apart, was used in analyses

Ethics statement:

All WHI participants provided informed consent and the study was approved by the Institutional Review Board (IRB) of the Fred Hutchinson Cancer Research Center.

WHI acknowledgements:

The WHI program is funded by the National Heart, Lung, and Blood Institute, National Institutes of Health, U.S. Department of Health and Human Services through contracts 75N92021D00001, 75N92021D00002, 75N92021D00003, 75N92021D00004, 75N92021D00005.

MESA

The Multi-Ethnic Study of Atherosclerosis (dbGaP accession phs000209) is a study of the characteristics of subclinical cardiovascular disease (disease detected non-invasively before it

has produced clinical signs and symptoms) and the risk factors that predict progression to clinically overt cardiovascular disease or progression of the subclinical disease [23]. MESA consisted of a diverse, population-based sample of an initial 6,814 asymptomatic men and women aged 45-84. 38 percent of the recruited participants were white, 28 percent African American, 22 percent Hispanic, and 12 percent Asian, predominantly of Chinese descent. Participants were recruited from six field centers across the United States: Wake Forest University, Columbia University, Johns Hopkins University, University of Minnesota, Northwestern University and University of California - Los Angeles. Participants are being followed for identification and characterization of cardiovascular disease events, including acute myocardial infarction and other forms of coronary heart disease (CHD), stroke, and congestive heart failure; for cardiovascular disease interventions; and for mortality. The first examination took place over two years, from July 2000 - July 2002. It was followed by five examination periods that were 17-20 months in length. Participants have been contacted every 9 to 12 months throughout the study to assess clinical morbidity and mortality.

Blood pressure measurement methods:

Resting BP was taken three times in the seated position after a five-minute rest using a Dinamap model Pro 100 automated oscillometric sphygmomanometer (Critikon, Tampa, Florida) [24] with the average of the last two measurements recorded and verified.

Ethics statements:

All MESA participants provided written informed consent, and the study was approved by the Institutional Review Boards at The Lundquist Institute (formerly Los Angeles BioMedical Research Institute) at Harbor-UCLA Medical Center, University of Washington, Wake Forest School of Medicine, Northwestern University, University of Minnesota, Columbia University, and Johns Hopkins University.

MESA acknowledgements:

MESA and the MESA SHARe projects are conducted and supported by the National Heart, Lung, and Blood Institute (NHLBI) in collaboration with MESA investigators. Support for MESA is provided by contracts 75N92020D00001, HHSN268201500003I, N01-HC-95159, 75N92020D00005, N01-HC-95160, 75N92020D00002, N01-HC-95161, 75N92020D00003, N01-HC-95162, 75N92020D00006, N01-HC-95163, 75N92020D00004, N01-HC-95164, 75N92020D00007, N01-HC-95165, N01-HC-95166, N01-HC-95167, N01-HC-95168, N01-HC-95169, UL1-TR-000040, UL1-TR-001079, and UL1-TR-001420, UL1TR001881, DK063491, and R01HL105756. MESA Family is conducted and supported by the National Heart, Lung, and Blood Institute (NHLBI) in collaboration with MESA investigators. Support is provided by grants and contracts R01HL071051, R01HL071205, R01HL071250, R01HL071251, R01HL071258, R01HL071259, and by the National Center for Research Resources, Grant UL1RR033176. The authors thank the other investigators, the staff, and the participants of the MESA study for their valuable contributions. A fill list of participating MESA investigators and institutes can be found at http://www.mesa-nhlbi.org.

MGB Biobank

Samples, genomic data, and health information were obtained from the Mass General Brigham (MGB) Biobank, a biorepository of consented patient samples at Mass General Brigham.

DNA samples

DNA samples are processed from whole blood that was collected as a dedicated research draw or as a clinical discard. Dedicated research samples are aimed to be processed within four hours of collection. Clinical discards are processed 24+ hours after collection. Whole blood is spun to buffy coat with a centrifuge and the buffy coat is stored in a freezer up to several months. The buffy coat is then extracted to DNA. The DNA is then placed in an ultralow freezer (-80ºC).Each DNA aliquot contains a minimum of 2 ug of DNA. The concentration varies.

Genotyping

Samples have been genotyped using three versions of the biobank SNP array offered by Illumina that is designed to capture the diversity of genetic backgrounds across the globe. The first batch of data was generated on the Multi-Ethnic Genotyping Array (MEGA) array, the first release of this SNP array. The second, third, and fourth batches were generated on the Expanded Multi-Ethnic Genotyping Array (MEGA Ex) array. All remaining data were generated on the Multi-Ethnic Global (MEG) BeadChip.

Imputation

Prior to performing imputation, files were converted to VCF format, separated by chromosomes. When multiple probes measured the same genotypes, they were checked for concordance and were set to a missing value if the genotypes did not match. Files were uploaded to the Michigan Imputation Server, and Genotypes were imputed using TOPMed reference panel. Genomic coordinates are provided in GRCh38.

Quality control

We performed quality control using PLINK (v2.0. We filtered SNPs with low-quality imputation ($r < 0.5$), with missing call rates > 0.1, HWE p-value less than $1x10^{-6}$ and MAF <1%.

We computed principal component (PC) using PLINK: we pruned the genotype data using a window size of 1000 variants, sliding across the genome with a step size of 250 variants at a time, filtering out any SNPs with LD R^2>0.1. We used unrelated individuals (3rd degree, identified using PLINK) to compute the loadings for the first 10 PCs.

PRS construction

We constructed PRS using PRSice 2, using the same SNPs as those based on clumping performed on the TOPMed dataset, and otherwise the same methodology. Including, for comparability, when scaling PRS by mean and SD we used the ones estimated on the TOPMed dataset from stage 2.

Curated Disease Populations

We used outcomes from "curated disease populations". These phenotypes were developed by the Biobank Portal team using both structured and unstructured electronic medical record (EMR) data and clinical, computational and statistical methods. Natural Language Processing (NLP) was used to extract data from narrative text. Chart reviews by disease experts helped identify features and variables associated with particular phenotypes and were also used to validate results of the algorithms. The process produced robust phenotype algorithms that were evaluated using metrics such as sensitivity, the proportion of true positives correctly identified as such, and positive predictive value (PPV), the proportion of individuals classified as cases by the algorithm [25]. The high throughput phenotyping algorithm is as follows:

1. Create an initial phenotype definition using ICD-9 diagnosis codes.
2. Broaden the definition by determining the most up-to-date features (comorbidities, symptoms, medications) that create a more accurate profile of the phenotype when combined with ICD-9 codes. Features are extracted from online medical literature and knowledge bases via an Automated Feature Extraction Protocol (AFEP).
3. Narrow and refine the definition by determining the features that occur most often in the Biobank data. Extract, code, and rank features contained in clinical narratives with Natural Language Processing (NLP).
4. Create a gold-standard patient set for training the method. Query coded EMR data for the set of patients having at least one ICD-9 code for the phenotype. Apply a statistical sampling algorithm to select a random subset of those patients for full chart review. A clinical expert performs a full chart review to classify the patients as positive or negative for the phenotype.

5. Train a statistical model that incorporates all features in the definition to predict the presence or absence of the phenotype against the gold-standard patient set.
6. Apply the trained model to the entire Biobank Population.

The following table provides the prevalence and AUC of the phenotypes that we used (AUC was computed based on step 4 above).

**Phenotype**	**Biobank Prevalence**	**AUC**
Coronary Artery Disease	13.5%	0.989
Hypertension	42.0%	0.912
Ischemic Stroke	3.1%	0.907
Type-II Diabetes Mellitus	10.6%	0.977
Obesity	48.9%	0.948

Association analyses with disease outcomes

We used the outcomes above in association analyses. We adjusted for current age, sex, genotype batch, and the first 10 PCs. We only used unrelated individuals in the analysis and therefore used standard logistic regression models.

Ethics statement

All Biobank subjects have provided their consent to join the MGB Biobank, which includes agreeing to provide a blood sample linked to the electronic medical record. Subjects also agree to be recontacted by the Partners Biobank staff as needed.

Acknowledgements

We thank Mass General Brigham Biobank for providing samples, genomic data, and health information data.

Supplementary Note 4: TOPMed and CCDG acknowledgements

Molecular data for the Trans-Omics in Precision Medicine (TOPMed) program was supported by the National Heart, Lung and Blood Institute (NHLBI). Genome sequencing for “NHLBI TOPMed - NHGRI CCDG: The BioMe Biobank at Mount Sinai” (phs001644) were performed at the Baylor College of Medicine Human Genome Sequencing Center and at the McDonnell Genome Institute (3UM1HG008853- WGS 01S2, HHSN26820160003 WGS 3I, HHSN26820160003 WGS 7I). Genome sequencing for “NHLBI TOPMed: Whole Genome Sequencing and Related Phenotypes in the Framingham Heart Study” (phs000974.v4.p3) was performed at the Broad Institute Genomics Platform (3R01HL092577-06S1, 3U54HG003067-12S2). DNA extraction for “NHLBI TOPMed: Genetic Epidemiology Network of Arteriopathy” (phs001345.v2.p1) was performed at the Mayo Clinic Genotyping Core and Genome sequencing was performed at the Broad Institute Genomics Platform (HHSN268201500014C) and the Northwest Genomics Center (3R01HL055673-18S1). Genome sequencing for “NHLBI TOPMed: The Jackson Heart Study” (phs000964.v1.p1) was performed at the Northwest Genomics Center (HHSN268201100037C).

Genome sequencing for the “NHLBI TOPMed: The Atherosclerosis Risk in Communities Study” (phs001211.v3.p2) was performed at the Broad Institute Genomics Platform (3R01HL092577-06S1) and the Baylor College of Medicine Human Genome Sequencing Center (HHSN268201500015C, 3U54HG003273-12S2). Genomics sequencing for “NHLBI TOPMed: Cardiovascular Health Study” (phs001368.v2.p1) was performed at the Baylor College of Medicine Human Genome Sequencing Center (3U54HG003273-12S2, HHSN268201500015C, HHSN268201600033I). Genome sequencing for “NHLBI TOPMed: Hispanic Community Health Study/Study of Latinos” (phs001395.v1.p1) was performed at the Baylor College of Medicine Human Genome Sequencing Center (HHSN268201600033I). Genome sequencing for “NHLBI TOPMed: Women’s Health Initiative (WHI)” (phs001237.v2.p1) was performed at the Broad Institute of MIT and Harvard (HHSN268201500014C). Genome sequencing for “NHLBI TOPMed: Multi-Ethnic Study of Atherosclerosis” (phs001416.v2.p1) was performed at the Baylor College of Medicine Human Genome Sequencing Center (HHSN268201500015C and 3U54HG003273-12S2) and the Broad Institute for MIT and Harvard (3R01HL092577-06S1). Genome Sequencing for the NHLBI TOPMed: CARDIA Study (phs001612) was performed at the Baylor College of Medicine Human genome Sequencing Center (contract HHSN268201600033I). Core support including centralized genomic read mapping and genotype calling, along with variant quality metrics and filtering were provided by the TOPMed Informatics Research Center (3R01HL-117626-02S1; contract HHSN268201800002I). Core support including phenotype harmonization, data management, sample-identity QC, and general program coordination were provided by the TOPMed Data Coordinating Center (R01HL-120393; U01HL-120393; contract HHSN268201800001I). We gratefully acknowledge the studies and participants who provided

biological samples and data for TOPMed. The Genome Sequencing Program (GSP) was funded by the National Human Genome Research Institute (NHGRI), the National Heart, Lung, and Blood Institute (NHLBI), and the National Eye Institute (NEI). The GSP Coordinating Center (U24 HG008956) contributed to cross-program scientific initiatives and provided logistical and general study coordination. The Centers for Common Disease Genomics (CCDG) program was supported by NHGRI and NHLBI, and whole genome sequencing was performed at the Baylor College of Medicine Human Genome Sequencing Center (UM1 HG008898 and R01HL059367).

Supplementary Note 5: TOPMed consortium authors

Namiko Abe[1], Gonçalo Abecasis[2], Francois Aguet[3], Christine Albert[4], Laura Almasy[5], Alvaro Alonso[6], Seth Ament[7], Peter Anderson[8], Pramod Anugu[9], Deborah Applebaum-Bowden[10], Kristin Ardlie[3], Dan Arking[11], Donna K Arnett[12], Allison Ashley-Koch[13], Stella Aslibekyan[14], Tim Assimes[15], Paul Auer[16], Dimitrios Avramopoulos[11], Najib Ayas[17], Adithya Balasubramanian[18], John Barnard[19], Kathleen Barnes[20], R. Graham Barr[21], Emily Barron-Casella[11], Lucas Barwick[22], Terri Beaty[11], Gerald Beck[23], Diane Becker[24], Lewis Becker[11], Rebecca Beer[25], Amber Beitelshees[7], Emelia Benjamin[26], Takis Benos[27], Marcos Bezerra[28], Larry Bielak[2], Joshua Bis[29], Thomas Blackwell[2], John Blangero[30], Eric Boerwinkle[31], Donald W. Bowden[32], Russell Bowler[33], Jennifer Brody[8], Ulrich Broeckel[34], Jai Broome[8], Deborah Brown[35], Karen Bunting[1], Esteban Burchard[36], Carlos Bustamante[37], Erin Buth[38], Brian Cade[39], Jonathan Cardwell[40], Vincent Carey[41], Julie Carrier[42], April Carson[43], Cara Carty[44], Richard Casaburi[45], Juan P Casas Romero[46], James Casella[11], Peter Castaldi[47], Mark Chaffin[3], Christy Chang[7], Yi-Cheng Chang[48], Daniel Chasman[49], Sameer Chavan[40], Bo-Juen Chen[1], Wei-Min Chen[50], Yii-Der Ida Chen[51], Michael Cho[41], Seung Hoan Choi[3], Lee-Ming Chuang[52], Mina Chung[53], Ren-Hua Chung[54], Clary Clish[55], Suzy Comhair[56], Matthew Conomos[38], Elaine Cornell[57], Adolfo Correa[58], Carolyn Crandall[45], James Crapo[59], L. Adrienne Cupples[60], Joanne Curran[61], Jeffrey Curtis[62], Brian Custer[63], Coleen Damcott[7], Dawood Darbar[64], Sean David[65], Colleen Davis[8], Michelle Daya[40], Mariza de Andrade[66], Lisa de las Fuentes[67], Paul de Vries[68], Michael DeBaun[69], Ranjan Deka[70], Dawn DeMeo[41], Scott Devine[7], Huyen Dinh[18], Harsha Doddapaneni[18], Qing Duan[71], Shannon Dugan-Perez[18], Ravi Duggirala[72], Jon Peter Durda[57], Susan K. Dutcher[73], Charles Eaton[74], Lynette Ekunwe[9], Adel El Boueiz[75], Patrick Ellinor[76], Leslie Emery[8], Serpil Erzurum[19], Charles Farber[50], Jesse Farek[18], Tasha Fingerlin[77], Matthew Flickinger[2], Myriam Fornage[31], Nora Franceschini[78], Chris Frazar[8], Mao Fu[7], Stephanie M. Fullerton[8], Lucinda Fulton[79], Stacey Gabriel[3], Weiniu Gan[25], Shanshan Gao[40], Yan Gao[9], Margery Gass[80], Heather Geiger[81], Bruce Gelb[82], Mark Geraci[27], Soren Germer[1], Robert Gerszten[83], Auyon Ghosh[41], Richard Gibbs[18], Chris Gignoux[15], Mark Gladwin[27], David Glahn[84], Stephanie Gogarten[8], Da-Wei Gong[7], Harald Goring[85], Sharon Graw[86], Kathryn J. Gray[87], Daniel Grine[40], Colin Gross[2], C. Charles Gu[79], Yue Guan[7], Xiuqing Guo[51], Namrata Gupta[3], David M. Haas[88], Jeff Haessler[80], Michael Hall[89], Yi Han[18], Patrick Hanly[90], Daniel Harris[91], Nicola L. Hawley[92], Jiang He[93], Ben Heavner[38], Susan Heckbert[94], Ryan Hernandez[36], David Herrington[95], Craig Hersh[96], Bertha Hidalgo[14], James Hixson[31], Brian Hobbs[41], John Hokanson[40], Elliott Hong[7], Karin Hoth[97], Chao (Agnes) Hsiung[98], Jianhong Hu[18], Yi-Jen Hung[99], Haley Huston[100], Chii Min Hwu[101], Marguerite Ryan Irvin[14], Rebecca Jackson[102], Deepti Jain[8], Cashell Jaquish[103], Jill Johnsen[104], Andrew Johnson[25], Craig Johnson[8], Rich Johnston[6], Kimberly Jones[11], Hyun Min Kang[105], Robert Kaplan[106], Sharon Kardia[2], Shannon Kelly[36], Eimear Kenny[82], Michael Kessler[7], Alyna Khan[8], Ziad Khan[18], Wonji Kim[107], John Kimoff[108], Greg Kinney[109], Barbara Konkle[110], Charles Kooperberg[80], Holly Kramer[111], Christoph Lange[112], Ethan Lange[40], Leslie Lange[113], Cathy Laurie[8], Cecelia Laurie[8], Meryl LeBoff[41], Jiwon Lee[41], Sandra Lee[18], Wen-Jane Lee[101], Jonathon LeFaive[2], David Levine[8], Dan Levy[25], Joshua Lewis[7], Xiaohui Li[51], Yun Li[71], Henry Lin[51], Honghuang Lin[114], Xihong Lin[115], Simin Liu[116], Yongmei Liu[117], Yu Liu[118], Ruth J.F. Loos[119], Steven Lubitz[76], Kathryn Lunetta[114], James Luo[25], Ulysses Magalang[120], Michael Mahaney[61], Barry

Make[11], Ani Manichaikul[50], Alisa Manning[121], JoAnn Manson[41], Lisa Martin[122], Melissa Marton[81], Susan Mathai[40], Rasika Mathias[11], Susanne May[38], Patrick McArdle[7], Merry-Lynn McDonald[123], Sean McFarland[107], Stephen McGarvey[124], Daniel McGoldrick[125], Caitlin McHugh[38], Becky McNeil[126], Hao Mei[9], James Meigs[127], Vipin Menon[18], Luisa Mestroni[86], Ginger Metcalf[18], Deborah A Meyers[128], Emmanuel Mignot[129], Julie Mikulla[25], Nancy Min[9], Mollie Minear[130], Ryan L Minster[27], Braxton D. Mitchell[7], Matt Moll[47], Zeineen Momin[18], May E. Montasser[7], Courtney Montgomery[131], Donna Muzny[18], Josyf C Mychaleckyj[50], Girish Nadkarni[82], Rakhi Naik[11], Take Naseri[132], Pradeep Natarajan[3], Sergei Nekhai[133], Sarah C. Nelson[38], Bonnie Neltner[40], Caitlin Nessner[18], Deborah Nickerson[134], Osuji Nkechinyere[18], Kari North[71], Jeff O'Connell[135], Tim O'Connor[7], Heather Ochs-Balcom[136], Geoffrey Okwuonu[18], Allan Pack[137], David T. Paik[138], Nicholette Palmer[139], James Pankow[140], George Papanicolaou[25], Cora Parker[141], Gina Peloso[142], Juan Manuel Peralta[72], Marco Perez[15], James Perry[7], Ulrike Peters[143], Patricia Peyser[2], Lawrence S Phillips[6], Jacob Pleiness[2], Toni Pollin[7], Wendy Post[144], Julia Powers Becker[145], Meher Preethi Boorgula[40], Michael Preuss[82], Bruce Psaty[8], Pankaj Qasba[25], Dandi Qiao[41], Zhaohui Qin[6], Nicholas Rafaels[146], Laura Raffield[147], Mahitha Rajendran[18], Vasan S. Ramachandran[114], D.C. Rao[79], Laura Rasmussen-Torvik[148], Aakrosh Ratan[50], Susan Redline[47], Robert Reed[7], Catherine Reeves[149], Elizabeth Regan[59], Alex Reiner[150], Muagututiâ€˜a Sefuiva Reupena[151], Ken Rice[8], Stephen Rich[50], Rebecca Robillard[152], Nicolas Robine[81], Dan Roden[153], Carolina Roselli[3], Jerome Rotter[154], Ingo Ruczinski[11], Alexi Runnels[81], Pamela Russell[40], Sarah Ruuska[100], Kathleen Ryan[7], Ester Cerdeira Sabino[155], Danish Saleheen[21], Shabnam Salimi[156], Sejal Salvi[18], Steven Salzberg[11], Kevin Sandow[157], Vijay G. Sankaran[158], Jireh Santibanez[18], Karen Schwander[79], David Schwartz[40], Frank Sciurba[27], Christine Seidman[159], Jonathan Seidman[160], FrÃ©dÃ©ric SÃ©riÃ¨s[161], Vivien Sheehan[162], Stephanie L. Sherman[163], Amol Shetty[7], Aniket Shetty[40], Wayne Hui-Heng Sheu[101], M. Benjamin Shoemaker[164], Brian Silver[165], Edwin Silverman[41], Robert Skomro[166], Albert Vernon Smith[167], Jennifer Smith[2], Josh Smith[8], Nicholas Smith[94], Tanja Smith[1], Sylvia Smoller[106], Beverly Snively[168], Michael Snyder[15], Tamar Sofer[41], Nona Sotoodehnia[8], Adrienne M. Stilp[8], Garrett Storm[169], Elizabeth Streeten[7], Jessica Lasky Su[170], Yun Ju Sung[79], Jody Sylvia[41], Adam Szpiro[8], Daniel Taliun[2], Hua Tang[171], Margaret Taub[11], Kent D. Taylor[172], Matthew Taylor[86], Simeon Taylor[7], Marilyn Telen[13], Timothy A. Thornton[8], Machiko Threlkeld[173], Lesley Tinker[174], David Tirschwell[8], Sarah Tishkoff[175], Hemant Tiwari[176], Catherine Tong[177], Russell Tracy[178], Michael Tsai[140], Dhananjay Vaidya[11], David Van Den Berg[179], Peter VandeHaar[2], Scott Vrieze[140], Tarik Walker[40], Robert Wallace[97], Avram Walts[40], Fei Fei Wang[8], Heming Wang[180], Jiongming Wang[167], Karol Watson[45], Jennifer Watt[18], Daniel E. Weeks[27], Joshua Weinstock[105], Bruce Weir[8], Scott T Weiss[181], Lu-Chen Weng[76], Jennifer Wessel[182], Cristen Willer[62], Kayleen Williams[38], L. Keoki Williams[183], Carla Wilson[41], James Wilson[184], Lara Winterkorn[81], Quenna Wong[8], Joseph Wu[138], Huichun Xu[7], Lisa Yanek[11], Ivana Yang[40], Ketian Yu[2], Seyedeh Maryam Zekavat[3], Yingze Zhang[185], Snow Xueyan Zhao[59], Wei Zhao[186], Xiaofeng Zhu[187], Michael Zody[1], Sebastian Zoellner[2]

1 - New York Genome Center, New York, New York; 2 - University of Michigan, Ann Arbor, Michigan; 3 - Broad Institute, Cambridge, Massachusetts; 4 - Cedars Sinai, Boston, Massachusetts; 5 - Children's Hospital of Philadelphia, University of Pennsylvania, Philadelphia, Pennsylvania; 6 - Emory University, Atlanta, Georgia; 7 - University of Maryland, Baltimore, Maryland; 8 - University of Washington, Seattle, Washington; 9 - University of Mississippi, Jackson, Mississippi; 10 - National Institutes of Health, Bethesda, Maryland; 11 - Johns Hopkins University, Baltimore, Maryland; 12 - University of Kentucky, Lexington, Kentucky; 13 - Duke University, Durham, North Carolina; 14 - University of Alabama, Birmingham, Alabama; 15 - Stanford University, Stanford, California; 16 - Medical College of Wisconsin, Milwaukee, Wisconsin; 17 - Medicine, Providence Health Care, Vancouver; 18 - Baylor College of Medicine Human Genome Sequencing Center, Houston, Texas; 19 - Cleveland Clinic, Cleveland, Ohio; 20 - Tempus, University of Colorado Anschutz Medical Campus, Aurora, Colorado; 21 - Columbia University, New York, New York; 22 - LTRC, The Emmes Corporation, Rockville, Maryland; 23 - Quantitative Health Sciences, Cleveland Clinic, Cleveland, Ohio; 24 - Medicine, Johns Hopkins University, Baltimore, Maryland; 25 - National Heart, Lung, and Blood Institute, National Institutes of Health, Bethesda, Maryland; 26 - Boston University School of Medicine, Boston University, Massachusetts General Hospital, Boston, Massachusetts; 27 - University of Pittsburgh, Pittsburgh, Pennsylvania; 28 - FundaÃ§Ã£o de Hematologia e Hemoterapia de Pernambuco - Hemope, Recife; 29 - Cardiovascular Health Research Unit, Department of Medicine, University of Washington, Seattle, Washington; 30 - Human Genetics, University of Texas Rio Grande Valley School of Medicine, Brownsville, Texas; 31 - University of Texas Health at Houston, Houston, Texas; 32 - Department of Biochemistry, Wake Forest Baptist Health, Winston-Salem, North Carolina; 33 - National Jewish Health, National Jewish Health, Denver, Colorado; 34 - Pediatrics, Medical College of Wisconsin, Milwaukee, Wisconsin; 35 - Pediatrics, University of Texas Health at Houston, Houston, Texas; 36 - University of California, San Francisco, San Francisco, California; 37 - Biomedical

Data Science, Stanford University, Stanford, California; 38 - Biostatistics, University of Washington, Seattle, Washington; 39 - Brigham and Women's Hospital, Brigham & Women's Hospital, Boston, Massachusetts; 40 - University of Colorado at Denver, Denver, Colorado; 41 - Brigham & Women's Hospital, Boston, Massachusetts; 42 - University of Montreal; 43 - Medicine, University of Mississippi, Jackson, Mississippi; 44 - Washington State University, Pullman, Washington; 45 - University of California, Los Angeles, Los Angeles, California; 46 - Brigham & Women's Hospital; 47 - Medicine, Brigham & Women's Hospital, Boston, Massachusetts; 48 - National Taiwan University, Taipei; 49 - Division of Preventive Medicine, Brigham & Women's Hospital, Boston, Massachusetts; 50 - University of Virginia, Charlottesville, Virginia; 51 - Lundquist Institute, Torrance, California; 52 - National Taiwan University Hospital, National Taiwan University, Taipei; 53 - Cleveland Clinic, Cleveland Clinic, Cleveland, Ohio; 54 - National Health Research Institute Taiwan, Miaoli County; 55 - Metabolomics Platform, Broad Institute, Cambridge, Massachusetts; 56 - Immunity and Immunology, Cleveland Clinic, Cleveland, Ohio; 57 - University of Vermont, Burlington, Vermont; 58 - Population Health Science, University of Mississippi, Jackson, Mississippi; 59 - National Jewish Health, Denver, Colorado; 60 - Biostatistics, Boston University, Boston, Massachusetts; 61 - University of Texas Rio Grande Valley School of Medicine, Brownsville, Texas; 62 - Internal Medicine, University of Michigan, Ann Arbor, Michigan; 63 - Vitalant Research Institute, San Francisco, California; 64 - University of Illinois at Chicago, Chicago, Illinois; 65 - University of Chicago, Chicago, Illinois; 66 - Health Quantitative Sciences Research, Mayo Clinic, Rochester, Minnesota; 67 - Department of Medicine, Cardiovascular Division, Washington University in St Louis, St. Louis, Missouri; 68 - Human Genetics Center, Department of Epidemiology, Human Genetics, and Environmental Sciences, University of Texas Health at Houston, Houston, Texas; 69 - Vanderbilt University, Nashville, Tennessee; 70 - University of Cincinnati, Cincinnati, Ohio; 71 - University of North Carolina, Chapel Hill, North Carolina; 72 - University of Texas Rio Grande Valley School of Medicine, Edinburg, Texas; 73 - Genetics, Washington University in St Louis, St Louis, Missouri; 74 - Brown University, Providence, Rhode Island; 75 - Channing Division of Network Medicine, Harvard University, Cambridge, Massachusetts; 76 - Massachusetts General Hospital, Boston, Massachusetts; 77 - Center for Genes, Environment and Health, National Jewish Health, Denver, Colorado; 78 - Epidemiology, University of North Carolina, Chapel Hill, North Carolina; 79 - Washington University in St Louis, St Louis, Missouri; 80 - Fred Hutchinson Cancer Research Center, Seattle, Washington; 81 - New York Genome Center, New York City, New York; 82 - Icahn School of Medicine at Mount Sinai, New York, New York; 83 - Beth Israel Deaconess Medical Center, Boston, Massachusetts; 84 - Department of Psychiatry, Boston Children's Hospital, Harvard Medical School, Boston, Massachusetts; 85 - University of Texas Rio Grande Valley School of Medicine, San Antonio, Texas; 86 - University of Colorado Anschutz Medical Campus, Aurora, Colorado; 87 - Obstetrics and Gynecology, Mass General Brigham, Boston, Massachusetts; 88 - OB/GYN, Indiana University, Indianapolis, Indiana; 89 - Cardiology, University of Mississippi, Jackson, Mississippi; 90 - Medicine, University of Calgary, Calgary; 91 - Genetics, University of Maryland, Philadelphia, Pennsylvania; 92 - Department of Chronic Disease Epidemiology, Yale University, New Haven, Connecticut; 93 - Tulane University, New Orleans, Louisiana; 94 - Epidemiology, University of Washington, Seattle, Washington; 95 - Wake Forest Baptist Health, Winston-Salem, North Carolina; 96 - Channing Division of Network Medicine, Brigham & Women's Hospital, Boston, Massachusetts; 97 - University of Iowa, Iowa City, Iowa; 98 - Institute of Population Health Sciences, NHRI, National Health Research Institute Taiwan, Miaoli County; 99 - Tri-Service General Hospital National Defense Medical Center; 100 - Blood Works Northwest, Seattle, Washington; 101 - Taichung Veterans General Hospital Taiwan, Taichung City; 102 - Internal Medicine, DIvision of Endocrinology, Diabetes and Metabolism, Oklahoma State University Medical Center, Columbus, Ohio; 103 - NHLBI, National Heart, Lung, and Blood Institute, National Institutes of Health, Bethesda, Maryland; 104 - Research Institute, Blood Works Northwest, Seattle, Washington; 105 - Biostatistics, University of Michigan, Ann Arbor, Michigan; 106 - Albert Einstein College of Medicine, New York, New York; 107 - Harvard University, Cambridge, Massachusetts; 108 - McGill University, MontrÃ©al; 109 - Epidemiology, University of Colorado at Denver, Aurora, Colorado; 110 - Medicine, Blood Works Northwest, Seattle, Washington; 111 - Public Health Sciences, Loyola University, Maywood, Illinois; 112 - Biostats, Harvard School of Public Health, Boston, Massachusetts; 113 - Medicine, University of Colorado at Denver, Aurora, Colorado; 114 - Boston University, Boston, Massachusetts; 115 - Harvard School of Public Health, Boston, Massachusetts; 116 - Epidemiology and Medicine, Brown University, Providence, Rhode Island; 117 - Cardiology, Duke University, Durham, North Carolina; 118 - Cardiovascular Institute, Stanford University, Stanford, California; 119 - The Charles Bronfman Institute for Personalized Medicine, Icahn School of Medicine at Mount Sinai, New York, New York; 120 - Division of Pulmonary, Critical Care and Sleep Medicine, Ohio State University, Columbus, Ohio; 121 - Broad Institute, Harvard University, Massachusetts General Hospital; 122 - cardiology, George Washington University,

Washington, District of Columbia; 123 - University of Alabama at Birmingham, University of Alabama, Birmingham, Alabama; 124 - Epidemiology, Brown University, Providence, Rhode Island; 125 - Genome Sciences, University of Washington, Seattle, Washington; 126 - RTI International; 127 - Medicine, Massachusetts General Hospital, Boston, Massachusetts; 128 - University of Arizona, Tucson, Arizona; 129 - Center For Sleep Sciences and Medicine, Stanford University, Palo Alto, California; 130 - National Institute of Child Health and Human Development, National Institutes of Health, Bethesda, Maryland; 131 - Genes and Human Disease, Oklahoma Medical Research Foundation, Oklahoma City, Oklahoma; 132 - Ministry of Health, Government of Samoa, Apia; 133 - Howard University, Washington, District of Columbia; 134 - Department of Genome Sciences, University of Washington, Seattle, Washington; 135 - University of Maryland, Balitmore, Maryland; 136 - University at Buffalo, Buffalo, New York; 137 - Division of Sleep Medicine/Department of Medicine, University of Pennsylvania, Philadelphia, Pennsylvania; 138 - Stanford Cardiovascular Institute, Stanford University, Stanford, California; 139 - Biochemistry, Wake Forest Baptist Health, Winston-Salem, North Carolina; 140 - University of Minnesota, Minneapolis, Minnesota; 141 - Biostatistics and Epidemiology Division, RTI International, Research Triangle Park, North Carolina; 142 - Department of Biostatistics, Boston University, Boston, Massachusetts; 143 - Fred Hutch and UW, Fred Hutchinson Cancer Research Center, Seattle, Washington; 144 - Cardiology/Medicine, Johns Hopkins University, Baltimore, Maryland; 145 - Medicine, University of Colorado at Denver, Denver, Colorado; 146 - CCPM, University of Colorado at Denver, Denver, Colorado; 147 - Genetics, University of North Carolina, Chapel Hill, North Carolina; 148 - Northwestern University, Chicago, Illinois; 149 - New York Genome Center, New York Genome Center, New York City, New York; 150 - Fred Hutchinson Cancer Research Center, University of Washington, Seattle, Washington; 151 - Lutia I Puava Ae Mapu I Fagalele, Apia; 152 - Sleep Research Unit, University of Ottawa Institute for Mental Health Research, University of Ottawa, Ottawa; 153 - Medicine, Pharmacology, Biomedicla Informatics, Vanderbilt University, Nashville, Tennessee; 154 - Pediatrics, Lundquist Institute, Torrance, California; 155 - Faculdade de Medicina, Universidade de Sao Paulo, Sao Paulo; 156 - Pathology, University of Maryland, Seattle, Washington; 157 - TGPS, Lundquist Institute, Torrance, California; 158 - Division of Hematology/Oncology, Harvard University, Boston, Massachusetts; 159 - Genetics, Harvard Medical School, Boston, Massachusetts; 160 - Harvard Medical School, Boston, Massachusetts; 161 - UniversitÃ© Laval, Quebec City; 162 - Pediatrics, Emory University, Atlanta, Georgia; 163 - Human Genetics, Emory University, Atlanta, Georgia; 164 - Medicine/Cardiology, Vanderbilt University, Nashville, Tennessee; 165 - UMass Memorial Medical Center, Worcester, Massachusetts; 166 - University of Saskatchewan, Saskatoon; 167 - University of Michigan; 168 - Biostatistical Sciences, Wake Forest Baptist Health, Winston-Salem, North Carolina; 169 - Genomic Cardiology, University of Colorado at Denver, Aurora, Colorado; 170 - Channing Department of Medicine, Brigham & Women's Hospital, Boston, Massachusetts; 171 - Genetics, Stanford University, Stanford, California; 172 - Institute for Translational Genomics and Populations Sciences, Lundquist Institute, Torrance, California; 173 - University of Washington, Department of Genome Sciences, University of Washington, Seattle, Washington; 174 - Cancer Prevention Division of Public Health Sciences, Fred Hutchinson Cancer Research Center, Seattle, Washington; 175 - Genetics, University of Pennsylvania, Philadelphia, Pennsylvania; 176 - Biostatistics, University of Alabama, Birmingham, Alabama; 177 - Department of Biostatistics, University of Washington, Seattle, Washington; 178 - Pathology & Laboratory Medicine, University of Vermont, Burlington, Vermont; 179 - USC Methylation Characterization Center, University of Southern California, University of Southern California, California; 180 - Brigham & Women's Hospital, Mass General Brigham, Boston, Massachusetts; 181 - Channing Division of Network Medicine, Department of Medicine, Brigham & Women's Hospital, Boston, Massachusetts; 182 - Epidemiology, Indiana University, Indianapolis, Indiana; 183 - Henry Ford Health System, Detroit, Michigan; 184 - Cardiology, Beth Israel Deaconess Medical Center, Cambridge, Massachusetts; 185 - Medicine, University of Pittsburgh, Pittsburgh, Pennsylvania; 186 - Department of Epidemiology, University of Michigan, Ann Arbor, Michigan; 187 - Department of Population and Quantitative Health Sciences, Case Western Reserve University, Cleveland, Ohio

References

1. Turley, P. *et al.* Multi-trait analysis of genome-wide association summary statistics using MTAG. *Nat. Genet.* **50,** 229–237 (2018).

2. Maier, R. M. *et al.* Improving genetic prediction by leveraging genetic correlations among human diseases and traits. *Nat. Commun.* **9,** 989 (2018).
3. Bulik-Sullivan, B. *et al.* An atlas of genetic correlations across human diseases and traits. *Nat. Genet.* **47,** 1236–1241 (2015).
4. Chang, C. C. *et al.* Second-generation PLINK: rising to the challenge of larger and richer datasets. *Gigascience* **4,** 7 (2015).
5. Yang, J., Lee, S. H., Goddard, M. E. & Visscher, P. M. GCTA: a tool for genome-wide complex trait analysis. *Am. J. Hum. Genet.* **88,** 76–82 (2011).
6. Maples, B. K., Gravel, S., Kenny, E. E. & Bustamante, C. D. RFMix: a discriminative modeling approach for rapid and robust local-ancestry inference. *Am. J. Hum. Genet.* **93,** 278–288 (2013).
7. Kanai, M. *et al.* Genetic analysis of quantitative traits in the Japanese population links cell types to complex human diseases. *Nat. Genet.* **50,** 390–400 (2018).
8. Evangelou, E. *et al.* Genetic analysis of over 1 million people identifies 535 new loci associated with blood pressure traits. *Nat. Genet.* **50,** 1412–1425 (2018).
9. The ARIC Investigators. The Atherosclerosis Risk in Communities (ARIC) Study: design and objectives. . *Am. J. Epidemiol.* **129,** 687–702 (1989).
10. Fried, L. P. *et al.* The Cardiovascular Health Study: design and rationale. *Ann Epidemiol* **1,** 263–276 (1991).
11. Friedman, G. D. *et al.* CARDIA: study design, recruitment, and some characteristics of the examined subjects. *J. Clin. Epidemiol.* **41,** 1105–1116 (1988).
12. Dawber, T. R., Kannel, W. B. & Lyell, L. P. An approach to longitudinal studies in a community: the Framingham Study. *Ann. N. Y. Acad. Sci.* **107,** 539–556 (1963).
13. Splansky, G. L. *et al.* The Third Generation Cohort of the National Heart, Lung, and Blood Institute's Framingham Heart Study: design, recruitment, and initial examination. *Am. J. Epidemiol.* **165,** 1328–1335 (2007).
14. Kannel, W. B., Feinleib, M., McNamara, P. M., Garrison, R. J. & Castelli, W. P. An investigation of coronary heart disease in families. The Framingham offspring study. *Am. J. Epidemiol.* **110,** 281–290 (1979).
15. Lavange, L. M. *et al.* Sample design and cohort selection in the Hispanic Community Health Study/Study of Latinos. *Ann Epidemiol* **20,** 642–649 (2010).
16. Sorlie, P. D. *et al.* Design and implementation of the Hispanic Community Health Study/Study of Latinos. *Ann Epidemiol* **20,** 629–641 (2010).
17. Sorlie, P. D. *et al.* Prevalence of hypertension, awareness, treatment, and control in the Hispanic Community Health Study/Study of Latinos. *Am. J. Hypertens.* **27,** 793–800 (2014).
18. Wyatt, S. B. *et al.* A community-driven model of research participation: the Jackson Heart Study Participant Recruitment and Retention Study. *Ethn Dis* **13,** 438–455 (2003).
19. Taylor, H. A. *et al.* Toward resolution of cardiovascular health disparities in African Americans: design and methods of the Jackson Heart Study. *Ethn Dis* **15,** S6-4 (2005).
20. Seals, S. R. *et al.* Calibration of blood pressure measurements in the Jackson Heart Study. *Blood Press Monit* **24,** 130–136 (2019).
21. Hays, J. *et al.* The Women's Health Initiative recruitment methods and results. *Ann Epidemiol* **13,** S18-77 (2003).
22. Design of the Women's Health Initiative clinical trial and observational study. The Women's

Health Initiative Study Group. *Control. Clin. Trials* **19,** 61–109 (1998).
23. Bild, D. E. *et al.* Multi-Ethnic Study of Atherosclerosis: objectives and design. *Am. J. Epidemiol.* **156,** 871–881 (2002).
24. Ramsey, M. Blood pressure monitoring: automated oscillometric devices. *J Clin Monit* **7,** 56–67 (1991).
25. Yu, S. *et al.* Toward high-throughput phenotyping: unbiased automated feature extraction and selection from knowledge sources. *J. Am. Med. Inform. Assoc.* **22,** 993–1000 (2015).
