# Supplementary material for: A multi-ethnic polygenic risk score is associated with hypertension prevalence and progression throughout adulthood
Source: Nat Commun. 2022 Jun 21;13:3549. doi: 10.1038/s41467-022-31080-2 (PMC9213527; doi:10.1038/s41467-022-31080-2)
Supplement: Supplementary file 3 — Reporting Summary [file 41467_2022_31080_MOESM3_ESM.pdf]

nature portfolio

Corresponding author(s): Tamar Sofer

Last updated by author(s): Apr 21, 2022

Reporting Summary

Nature Portfolio wishes to improve the reproducibility of the work that we publish. This form provides structure for consistency and transparency in reporting. For further information on Nature Portfolio policies, see our Editorial Policies and the Editorial Policy Checklist.

Statistics

For all statistical analyses, confirm that the following items are present in the figure legend, table legend, main text, or Methods section.

n/a	Confirmed	
☐	☒	The exact sample size (*n*) for each experimental group/condition, given as a discrete number and unit of measurement
☐	☒	A statement on whether measurements were taken from distinct samples or whether the same sample was measured repeatedly
☐	☒	The statistical test(s) used AND whether they are one- or two-sided<br>*Only common tests should be described solely by name; describe more complex techniques in the Methods section.*
☐	☒	A description of all covariates tested
☐	☒	A description of any assumptions or corrections, such as tests of normality and adjustment for multiple comparisons
☐	☒	A full description of the statistical parameters including central tendency (e.g. means) or other basic estimates (e.g. regression coefficient) AND variation (e.g. standard deviation) or associated estimates of uncertainty (e.g. confidence intervals)
☐	☒	For null hypothesis testing, the test statistic (e.g. *F*, *t*, *r*) with confidence intervals, effect sizes, degrees of freedom and *P* value noted<br>*Give P values as exact values whenever suitable.*
☒	☐	For Bayesian analysis, information on the choice of priors and Markov chain Monte Carlo settings
☒	☐	For hierarchical and complex designs, identification of the appropriate level for tests and full reporting of outcomes
☐	☒	Estimates of effect sizes (e.g. Cohen's *d*, Pearson's *r*), indicating how they were calculated

Our web collection on statistics for biologists contains articles on many of the points above.

Software and code

Policy information about availability of computer code

Data collection	No software was used.
Data analysis	The following softwares were used: R/3.6.3, Python 2.7,PRSice 2.3.1.e , PLINK v1.9, PLINKv.2.0, ldsc v1.0.1, GCTA v1.91.1, MTAG(v1.0.8), METAL, SMTPRed, GENESIS R Packages v2.16.1, pROC R package v1.16.2, lme4 v1.1.23<br>Costume code can be found at : https://github.com/nkurniansyah/Hypertension_PRS

For manuscripts utilizing custom algorithms or software that are central to the research but not yet described in published literature, software must be made available to editors and reviewers. We strongly encourage code deposition in a community repository (e.g. GitHub). See the Nature Portfolio guidelines for submitting code & software for further information.

Data

Policy information about availability of data

All manuscripts must include a data availability statement. This statement should provide the following information, where applicable:

- Accession codes, unique identifiers, or web links for publicly available datasets
- A description of any restrictions on data availability
- For clinical datasets or third party data, please ensure that the statement adheres to our policy

TOPMed freeze 8 Whole Genome Sequencing (WGS) data are available under restricted access by application to dbGaP according to the study specific accessions: ARIC: "phs001416 [https://www.ncbi.nlm.nih.gov/projects/gap/cgi-bin/study.cgi?study_id=phs001416.v1.p1]", BioMe: "phs001644 [https://www.ncbi.nlm.nih.gov/projects/gap/cgi-bin/study.cgi?study_id=phs001644.v2.p2]", CARDIA: "phs001612 [https://www.ncbi.nlm.nih.gov/projects/gap/cgi-bin/study.cgi?study_id=phs001612.v1.p1]", CHS: "phs001368 [https://www.ncbi.nlm.nih.gov/projects/gap/cgi-bin/study.cgi?study_id=phs001368.v3.p2]", FHS: "phs000974 [https://www.ncbi.nlm.nih.gov/projects/gap/cgi-bin/study.cgi?study_id=phs000974.v4.p3]", GENOA: "phs001345 [https://www.ncbi.nlm.nih.gov/projects/gap/cgi-

bin/study.cgi?study_id=phs001345.v3.p1]", HCHS/SOL: "phs001395 [https://www.ncbi.nlm.nih.gov/projects/gap/cgi-bin/study.cgi?study_id=phs001395.v2.p1]", JHS: "phs000964 [https://www.ncbi.nlm.nih.gov/projects/gap/cgi-bin/study.cgi?study_id=phs000964.v5.p1]", MESA: "phs001211 [https://www.ncbi.nlm.nih.gov/projects/gap/cgi-bin/study.cgi?study_id=phs001211.v4.p3]", WHI: "phs001237 [https://www.ncbi.nlm.nih.gov/projects/gap/cgi-bin/study.cgi?study_id=phs001237.v3.p1]". Study phenotypes are available from dbGaP from study accession: ARIC: "phs000090 [https://www.ncbi.nlm.nih.gov/projects/gap/cgi-bin/study.cgi?study_id=phs000090.v7.p1]", BioMe: "phs001644 [https://www.ncbi.nlm.nih.gov/projects/gap/cgi-bin/study.cgi?study_id=phs001644.v2.p2]", CARDIA: "phs000285 [https://www.ncbi.nlm.nih.gov/projects/gap/cgi-bin/study.cgi?study_id=phs000285.v3.p2]", CHS: "phs000287 [https://www.ncbi.nlm.nih.gov/projects/gap/cgi-bin/study.cgi?study_id=phs000287.v7.p1]", FHS: "phs000007 [https://www.ncbi.nlm.nih.gov/projects/gap/cgi-bin/study.cgi?study_id=phs000007.v32.p13]", GENOA: "phs000379 [https://www.ncbi.nlm.nih.gov/projects/gap/cgi-bin/study.cgi?study_id=phs000379.v1.p1]", HCHS/SOL: "phs000810 [https://www.ncbi.nlm.nih.gov/projects/gap/cgi-bin/study.cgi?study_id=phs000810.v1.p1]", JHS: "phs000286 [https://www.ncbi.nlm.nih.gov/projects/gap/cgi-bin/study.cgi?study_id=phs000286.v6.p2]", MESA: "phs000209 [https://www.ncbi.nlm.nih.gov/projects/gap/cgi-bin/study.cgi?study_id=phs000209.v13.p3]", WHI: "phs000200 [https://www.ncbi.nlm.nih.gov/projects/gap/cgi-bin/study.cgi?study_id=phs000200.v12.p3]". The Summary statistics from the MVP BP GWAS are available from dbGaP by application to study accession "phs001672 [https://www.ncbi.nlm.nih.gov/projects/gap/cgi-bin/study.cgi?study_id=phs001672.v4.p1]". The summary statistics from the PAN-UKBB BP GWAS are available at https://pan.ukbb.broadinstitute.org. MGB Biobank genotyping and phenotypic data are available to Mass General Brigham investigators with required approval from the Mass General Brigham Institutional Review board (IRB). The data to construct the HTN-PRS generated in this study are available in the GitHub repository https://github.com/nkurniansyah/Hypertension_PRS. Source Data displayed in Figures 3-7 are provided with this paper.

Field-specific reporting

Please select the one below that is the best fit for your research. If you are not sure, read the appropriate sections before making your selection.

☒ Life sciences ☐ Behavioural & social sciences ☐ Ecological, evolutionary & environmental sciences

For a reference copy of the document with all sections, see nature.com/documents/nr-reporting-summary-flat.pdf

Life sciences study design

All studies must disclose on these points even when the disclosure is negative.

Sample size	The sample size was 89,550 individuals across multiple stage 1, stage 2, stage 3, and stage 4 datasets. We knew that these sample sizes are sufficient for the analysis because other published manuscripts performed associations analysis of blood pressure and hypertension traits with PRS and identified associations with smaller sample sizes.
Data exclusions	No exclusions: all individuals from the participating studies with phenotype values and appropriate consent were included.
Replication	We trained the PRS on stage 1 dataset, and tested it in the independent stage 2 dataset. We also validated the association again in the independent stage 4 dataset.
Randomization	There was no randomization because the data do not represent a trial, but rather an observational study.
Blinding	Because there was no trial/experiment, we analyzed observational data.

Reporting for specific materials, systems and methods

We require information from authors about some types of materials, experimental systems and methods used in many studies. Here, indicate whether each material, system or method listed is relevant to your study. If you are not sure if a list item applies to your research, read the appropriate section before selecting a response.

Materials & experimental systems

n/a	Involved in the study	
☒	☐	Antibodies
☒	☐	Eukaryotic cell lines
☒	☐	Palaeontology and archaeology
☒	☐	Animals and other organisms
☐	☒	Human research participants
☒	☐	Clinical data
☒	☐	Dual use research of concern

Methods

n/a	Involved in the study	
☒	☐	ChIP-seq
☒	☐	Flow cytometry
☒	☐	MRI-based neuroimaging

Human research participants

Policy information about studies involving human research participants

Population characteristics	See supplementary table 2, 3, and 6 for a detailed description of the research participants. Briefly,The TOPMed dataset included 52,436 individuals from 10 TOPMed. Based on the characteristics of these studies, the TOPMed data was split into stage 1, stage 2, and stage 3 datasets. Stage 1 dataset was BioMe, which included 10,314 diverse individuals with prevalent

hypertension status. Stage 2 dataset included 39,035 individuals from an additional eight studies, with all individuals being genetically unrelated (at the third degree) to those in the training dataset. Stage 2 dataset was longitudinal, with hypertension status assessed in two exams, on average 4-6 years apart. Individuals were self-reported from four predominant U.S. race/ethnic backgrounds, with 22,701 EA, 8,822 AA, 6,718 HA, and 794 AsA individuals. Stage 3 dataset included the CARDIA study, with 6 exams over 15 years follow up of young Black and White individuals, and was used to compare the development of hypertension risk within PRS strata across the two race/ethnic backgrounds. Stage 4 dataset was the MGB Biobank, including 40,201 unrelated individuals.

Recruitment	Multiple studies participated in the analyses in this manuscript. All the detail can be found in supplementary material in the study description section.

Ethics oversight

As public datasets available by application from the NHI dbGaP repository, all participants in TOPMed consortia studied provided informed consent to their parent studies at their recruitment site. All the details can be found in supplementary material in the study description section.

BioMe:
The BioMe cohort was approved by the Institutional Review Board at the Icahn School of Medicine at Mount Sinai. All BioMe participants provided written, informed consent for genomic data sharing.
ARIC:
The ARIC study has been approved by Institutional Review Boards (IRB) at all participating institutions: University of North Carolina at Chapel Hill IRB, Johns Hopkins University IRB, University of Minnesota IRB, and University of Mississippi Medical Center IRB. Study participants provided written informed consent at all study visits.
CHS:
All CHS participants provided informed consent, and the study was approved by the Institutional Review Board [or ethics review committee] of University Washington
CARDIA:
All CARDIA participants provided informed consent, and the study was approved by the Institutional Review Boards of the University of Alabama at Birmingham and the University of Texas Health Science Center at Houston.
FHS:
The Framingham Heart Study was approved by the Institutional Review Board of the Boston University Medical Center. All study participants provided written informed consent.
GENOA:
Written informed consent was obtained from all subjects and approval was granted by participating institutional review boards (University of Michigan, University of Mississippi Medical Center, and Mayo Clinic).
HCHS/SOL:
Ethics statement:
This study was approved by the institutional review boards (IRBs) at each field center, where all participants gave written informed consent, and by the Non-Biomedical IRB at the University of North Carolina at Chapel Hill, to the HCHS/SOL Data Coordinating Center. All IRBs approving the study are: Non-Biomedical IRB at the University of North Carolina at Chapel Hill. Chapel Hill, NC; Einstein IRB at the Albert Einstein College of Medicine of Yeshiva University. Bronx, NY; IRB at Office for the Protection of Research Subjects (OPRS), University of Illinois at Chicago. Chicago, IL; Human Subject Research Office, University of Miami. Miami, FL; Institutional Review Board of San Diego State University. San Diego, CA.
JHS:
The JHS study was approved by Jackson State University, Tougaloo College, and the University of Mississippi Medical Center IRBs, and all participants provided written informed consent.
WHI:
All WHI participants provided informed consent and the study was approved by the Institutional Review Board (IRB) of the Fred Hutchinson Cancer Research Center.
MESA:
All MESA participants provided written informed consent, and the study was approved by the Institutional Review Boards at The Lundquist Institute (formerly Los Angeles BioMedical Research Institute) at Harbor-UCLA Medical Center, University of Washington, Wake Forest School of Medicine, Northwestern University, University of Minnesota, Columbia University, and Johns Hopkins University.
MGB Biobank:
All Biobank subjects have provided their consent to join the MGB Biobank, which includes agreeing to provide a blood sample linked to the electronic medical record. Subjects also agree to be recontacted by the Partners Biobank staff as needed.

Note that full information on the approval of the study protocol must also be provided in the manuscript.
